# NUP-1 Is a Large Coiled-Coil Nucleoskeletal Protein in Trypanosomes with Lamin-Like Functions

**DOI:** 10.1371/journal.pbio.1001287

**Published:** 2012-03-27

**Authors:** Kelly N. DuBois, Sam Alsford, Jennifer M. Holden, Johanna Buisson, Michal Swiderski, Jean-Mathieu Bart, Alexander V. Ratushny, Yakun Wan, Philippe Bastin, J. David Barry, Miguel Navarro, David Horn, John D. Aitchison, Michael P. Rout, Mark C. Field

**Affiliations:** 1 Department of Pathology, University of Cambridge, Cambridge, United Kingdom; 2 London School of Hygiene & Tropical Medicine, London, United Kingdom; 3 Trypanosome Cell Biology Unit, Pasteur Institute and Centre National de la Recherche Scientifique, Paris, France; 4 Wellcome Trust Center for Molecular Parasitology, University of Glasgow, Glasgow, United Kingdom; 5 Instituto de Parasitología y Biomedicina López-Neyra, Consejo Superior de Investigaciones Cientificas, Granada, Spain; 6 The Institute for Systems Biology, Seattle, Washington, United States of America; 7 Seattle Biomedical Research Institute, Seattle, Washington, United States of America; 8 The Rockefeller University, New York, New York, United States of America; National Cancer Institute, United States of America

## Abstract

A unifying feature of eukaryotic nuclear organization is genome segregation into transcriptionally active euchromatin and transcriptionally repressed heterochromatin. In metazoa, lamin proteins preserve nuclear integrity and higher order heterochromatin organization at the nuclear periphery, but no non-metazoan lamin orthologues have been identified, despite the likely presence of nucleoskeletal elements in many lineages. This suggests a metazoan-specific origin for lamins, and therefore that distinct protein elements must compose the nucleoskeleton in other lineages. The trypanosomatids are highly divergent organisms and possess well-documented but remarkably distinct mechanisms for control of gene expression, including polycistronic transcription and *trans-*splicing. NUP-1 is a large protein localizing to the nuclear periphery of *Trypanosoma brucei* and a candidate nucleoskeletal component. We sought to determine if NUP-1 mediates heterochromatin organization and gene regulation at the nuclear periphery by examining the influence of NUP-1 knockdown on morphology, chromatin positioning, and transcription. We demonstrate that NUP-1 is essential and part of a stable network at the inner face of the trypanosome nuclear envelope, since knockdown cells have abnormally shaped nuclei with compromised structural integrity. NUP-1 knockdown also disrupts organization of nuclear pore complexes and chromosomes. Most significantly, we find that NUP-1 is required to maintain the silenced state of developmentally regulated genes at the nuclear periphery; NUP-1 knockdown results in highly specific mis-regulation of telomere-proximal silenced variant surface glycoprotein (*VSG*) expression sites and procyclin loci, indicating a disruption to normal chromatin organization essential to life-cycle progression. Further, NUP-1 depletion leads to increased *VSG* switching and therefore appears to have a role in control of antigenic variation. Thus, analogous to vertebrate lamins, NUP-1 is a major component of the nucleoskeleton with key roles in organization of the nuclear periphery, heterochromatin, and epigenetic control of developmentally regulated loci.

## Abstract 2

NUP1, the first example of a nuclear lamin analog in nonmetazoans, performs roles similar to those of lamins in maintaining the structure and organization of the nucleus in *Trypanosoma brucei*.

## Author Summary

Eukaryotes—fungi, plants, animals, and many unicellular organisms—are defined by the presence of a cell nucleus that contains the chromosomes and is enveloped by a lipid membrane lined on the inner face with a protein network called the lamina. Among other functions, the lamina serves as an anchorage site for the ends of chromosomes. In multicellular animals (metazoa), the lamina comprises a few related proteins called lamins, which are very important for many functions related to the nucleus; abnormal lamins result in multiple nuclear defects and diseases, including inappropriate gene expression and premature aging. Until now, however, lamins had been found only in metazoa; no protein of equivalent function had been identified in plants, fungi, or unicellular organisms. Here, we describe a protein from African trypanosomes—the single-cell parasites that cause sleeping sickness—that fulfils many lamin-like roles, including maintaining nuclear structure and organizing the chromosomes of this organism. We show that this protein, which we call NUP-1 for nuclear periphery protein-1, is vital for the antigenic variation mechanisms that allow the parasite to escape the host immune response. We propose that NUP-1 is a lamin analogue that performs similar functions in trypanosomes to those of authentic lamins in metazoa. These findings, we believe, have important implications for understanding the evolution of the nucleus.

## Introduction

Eukaryotic genomes are primarily organized as linear chromosomes and further segregated into transcriptionally active euchromatin and repressed heterochromatin [1]–[3]. In metazoa such chromatin organization requires the coiled-coil lamins, intermediate filament proteins that form a stable meshwork between the nuclear envelope (NE) and nuclear matrix, physically associating with peripheral heterochromatin [2],[4],[5]. Lamins directly participate in nuclear pore complex (NPC) positioning, maintenance of nuclear structure, spindle assembly, and control of developmental gene expression programs [6]–[9]. Lamins also function in positioning of the nucleus within the cell, and nuclear reassembly following mitotic NE vesiculation in open mitosis [10]–[13]. In humans, aberrant lamin protein structure or expression can lead to irregular nuclei and inappropriate gene expression, manifesting as pathological laminopathies, including progeria and muscular dystrophies [14]. Remarkably, no lamin orthologues had been identified in non-metazoa [15]. Moreover in yeasts, lamins and a major nucleoskeleton are clearly absent, despite the presence of apparent heterochromatin [16]. Together, this implies that the lamin-dependent mechanisms of heterochromatin organization in metazoan cells are a lineage-specific feature, and have evolved relatively recently, following the split between the animals and fungi [17].

Nevertheless, structures morphologically resembling nuclear peripheral heterochromatin and a lamina have been described in several divergent eukaryotic lineages, but their molecular basis has remained elusive [18]–[20]. For plants, for example, equivocal evidence is suggestive of the presence of a lamina-like nucleoskeletal structure (discussed in [21]). Further, heterochromatin is also tethered to the nuclear envelope of plants, and at least one candidate nucleoskeletal protein, NMP-1, has been identified. NMP-1 is a 36 kDa predominantly alpha-helical protein that associates with the nuclear matrix, but remains functionally uncharacterized, and is also likely plant specific [22].

Trypanosomatids are highly divergent organisms, whose origins may even lie close to the Eukaryotic root. Their mode of transcriptional control is highly unusual and, for the most part, independent of conventional promoter control, relying instead on polycistronic transcription and *trans*-splicing. Trypanosomes have structures reminiscent of a peripheral nucleoskeleton, while they also possess prominent heterochromatin-like material at the nuclear periphery that is implicated in control of gene expression [23]. The African trypanosome *T. brucei* is an obligate parasite living primarily in the blood, lymphatics, and cerebrospinal fluid when in the mammalian host (bloodstream form; BSF) and in the midgut and salivary glands in the Tsetse fly (procyclic form; PCF). The very different environments encountered by the parasite between these two hosts demand rapid and complex transcriptional changes. Further, the trypanosome cell cycle is highly coordinated, with precisely ordered division of organelles and suborganelles [24] and nuclear DNA elements. In trypanosomes these consist of 11 pairs of conventional megabase chromosomes harbouring the majority of protein coding genes plus dozens of unusual lower molecular weight minichromosomes containing mainly *VSG* genes. These two classes of chromosome segregate during mitosis with differential kinetics, location, and possibly also mechanism [25].

The BSF has a sophisticated system for immune evasion based on antigenic variation via expression at high copy number of a single variant surface glycoprotein (*VSG*). Periodic switching of the active *VSG* gene prevents elimination of the entire parasite population by allowing a subpopulation to escape the host immune response. *VSG* expression is tightly controlled to ensure monoallelic expression and takes place exclusively from telomere-proximal expression sites (ESs). An ES is present at many of the megabase chromosome telomeric regions. Further, *VSG* is developmentally regulated; the procyclic stage expresses only a second dominant surface protein, named procyclin. In contrast to higher eukaryotes, with largely promoter-controlled gene systems, trypanosome megabase chromosomes are organized into extensive polycistronic units, and mRNA levels are chiefly regulated by post-transcriptional mechanisms [26],[27]. However, trypanosomatids do possess chromatin subcompartments implicated in the control of gene expression. In *T. brucei*, electron dense heterochromatin encompassing telomeric regions is largely restricted to the nuclear periphery. Critically, in BSFs an RNA polymerase I–containing extranucleolar expression site body, located within the nuclear interior, provides an environment permissive for *VSG* transcription [28]–[31]. As telomeres carry multiple repressed *VSG* genes, expression site translocation between peripheral heterochromatin and the expression site body likely mediates antigenic variation [29], which therefore depends on chromatin organization and involves epigenetic mechanisms.

While several chromatin-remodelling and histone-modifying enzymes that act at telomeric regions have been described, no nucleoskeletal components acting specifically on trypanosome chromatin organization, with functions analogous to metazoan lamins, are known [32]–[34]. However, one candidate is NUP-1, a large coiled-coil protein that coenriches with the nuclear envelope and appears to be a component of fibrous material subtending the inner NE. We sought to determine if NUP-1 functions in heterochromatin organization and gene regulation at the nuclear periphery in *T. brucei*, which would have implications for the evolutionary origins of such mechanisms [23]. Using multiple approaches, we show that NUP-1 has several functions that are clearly analogous to those of metazoan lamins, including mediation of nuclear structural integrity, epigenetic chromatin organization, and maintenance of developmentally regulated gene expression. We propose that such mechanisms are likely widespread amongst eukaryotes, and hence an ancient and fundamental feature of gene control, rather than a lineage-specific aspect of metazoan cells.

## Results

### NUP-1 Is a Coiled-Coil Protein

**Figure 1:**
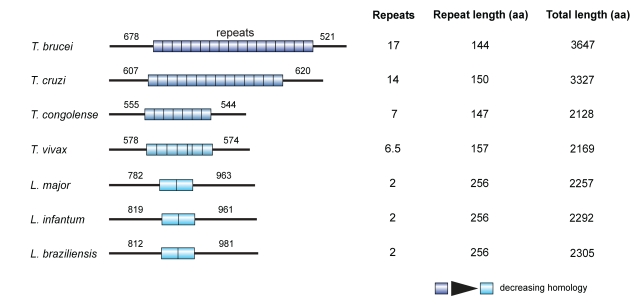
NUP-1 is a large coiled coil protein identified in *T. brucei* and restricted to trypanosomatids. A single NUP-1 orthologue is present in each trypanosomatid genome. Bars represent N- and C-terminal domains, cylinders α-helical repeats. Numbers above domains denote the number of amino acid residues predicted in each domain. Repeat number and length and total protein length in amino acids (aa) are also indicated.

NUP-1 is a large protein (pI 5.07, MW>400 kDa) associated with the nuclear periphery [23],[35] that was identified as a major component of the *T. brucei* NE proteome [36]. As predicted by both COILS and CCHMM_PROF, NUP-1 is almost exclusively coiled-coil with a central region of 17 near-perfect repeats of 144 amino acids (Figure 1A) [37],[38]. No *trans*-membrane helices were predicted. Moreover, the N- and C-terminal regions are also predicted to be predominantly alpha-helical and extended (>90% confidence, http://www.sbg.bio.ic.ac.uk/phyre2/); given that the rise per residue is ∼1.5 Å, this predicts that were the structure fully extended, the NUP-1 monomer would extend nearly to 400 nm, which is ∼25% of the diameter of the trypanosome nucleus.

A single NUP-1 syntenic orthologue was present in each trypanosomatid genome examined (Figure 1, Table S1), each exhibiting the same structure as NUP-1 but varying in size and number of repeats; the repeats within each NUP-1 orthologue are nearly identical but diverge significantly between species. Further, NUP-1 orthologues in *Trypanosoma* species diverge significantly from those in *Leishmania* (Figure 1, Table S2). So far, BLAST has failed to identify sequences with significant similarity to NUP-1 in *Phytomonas*, *Bodo saltans* (a free-living kinetoplastid), or *Euglena gracilis*.

### NUP-1 Localizes to the Nuclear Periphery within a Stable Lattice

**Figure 2:**
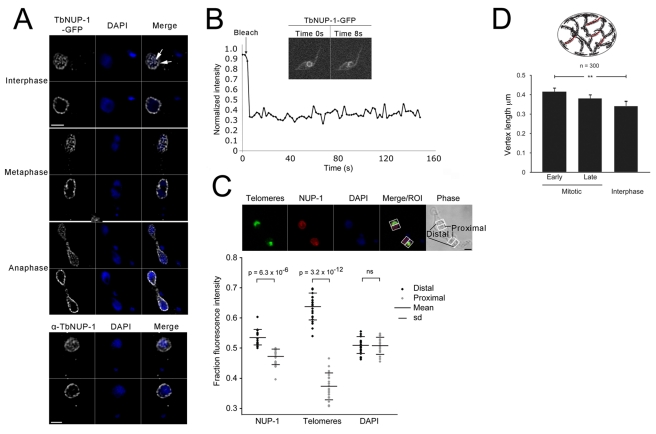
NUP-1 localizes to a stable network around the periphery of the nucleus. (A) Fixed PCF cells expressing NUP-1-GFP (white) or BSF cells probed with an anti-NUP-1 antibody (white) were imaged by confocal microscopy. Shown are optical sections of the edge and centre of nuclei taken from image series along the *z*-axis. DAPI was used to visualize the DNA (blue). Bar: 2 µm. Arrowheads at right panel indicate two putative puncta (i.e., foci of NUP-1 reactivity from which linear regions of NUP-1 reactivity emanate). See also Movies S1A–D. (B) FRAP of NUP-1-GFP. After bleaching a portion of the nucleus, no fluorescence recovery was observed during 150 s. See also Movie S5. (C) Top: Cells were probed with an anti-NUP-1 antibody (red) and FISH for telomeres (green). DAPI was used to visualize DNA. Nuclei of dividing cells were sectioned into proximal and distal halves for analysis based on the position of telomeres. Bar: 2 µm. Bottom: Fluorescence intensity in confocal nuclear sections of 21 dividing nuclei was recorded for NUP-1, telomeres, and DAPI and plotted as a fraction of the total fluorescence in each nucleus. Means and standard deviations (SD) are shown. The Student's paired *t* test was used to determine the *p* value of the fluorescence difference in the proximal compared to the distal half of the nucleus. NS, not significant. (D) Distance between NUP-1 puncta was measured using Metamorph software in cells that were in interphase (1K1N) or early or late mitotic (2K1N). Diagram at top illustrates the measurements taken, essentially of the inter-puncta distance (red). A total of 300 cells were analysed, and the statistical significance calculated using a Student's paired *t* test (*p*>0.01).

**Figure 3:**
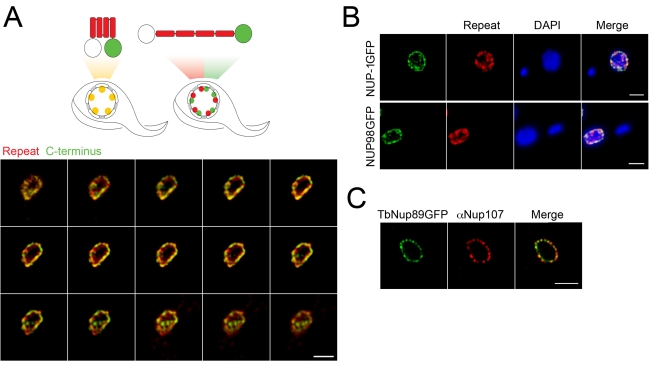
NUP-1 is arranged in an extended conformation. (A) Top: Schematic showing NUP-1 in either a compact (left) or an extended (right) conformation. Repeats are shown in red, and the GFP-tagged C-terminal domain is in green. The untagged N-terminus is white. Predicted staining patterns for each conformer are shown below the schematics. Lower: Cells expressing NUP-1-GFP were probed with anti-GFP (green) and anti-NUP-1 repeat region antibody (red) and imaged by confocal microscopy. A montage of 15 confocal *z*-stack slices through the nucleus of a single trypanosome is shown. There is clear discrimination between the red and green channels, consistent with an extended conformation. (B) Top: Cells expressing NUP-1-GFP were probed with an anti-GFP (green) antibody and a secondary antibody that was immunoabsorbed to eliminate cross-species reactivity and anti-NUP-1 repeat region antibody (red) and imaged by confocal microscopy. Shown is a central slice across the *z*-axis. DAPI was used to visualise DNA. Bar: 2 µm for all panels. In independent stains using either the anti-GFP or repeat region antibodies alone, it was clear that there was no cross-reactivity (unpublished data). Lower: Cells expressing TbNup98-GFP were probed with anti-GFP (green) and anti-NUP-1 repeat region antibody (red) and imaged by confocal microscopy. Shown is the central slice along the *z*-axis, also demonstrating clear separation of the nuclear pore complex from the NUP-1 repeat staining. (C) Cells expressing TbNup89-GFP were probed with anti-GFP (green) and an anti-FG repeat antibody (red) to visualize the NPCs and imaged by confocal microscopy. There is clear high correspondence between the red and green stains, consistent with previous data [36].

*NUP-1* mRNA is expressed at similar levels in BSF and PCF *T. brucei*, indicating a role throughout the life cycle (Figure S1). We established the location of NUP-1 by C-terminal genomic tagging of one allele with GFP in PCF cells followed by confocal microscopy. Fluorescence was observed at the nuclear periphery, and taken together with previous immunoEM, subcellular fractionation, and monoclonal antibody studies, indicates that NUP-1 is localized to a net-like structure at the nuclear periphery. The location described here has a more net-like distribution compared to the original description, which suggested a punctate nuclear rim localization, and was interpreted as a potential nucleoporin (Figures 2 and 3, Movies S1, S2, S3, and S4) [23],[35]. A similar pattern was seen in BSF cells with rabbit polyclonal antibodies raised to the NUP-1 repeat (Figure 2A, Movie S4), demonstrating that the GFP tag did not affect NUP-1 localization and confirming that the network is likely a more accurate view of NUP-1 distribution and not the puncta seen earlier [35]. We note that the vertex length (i.e., the distance between strongly stained puncta and apparent fibres or fibrils) is somewhat variable, but is similar to the 400 nm of the putative extended NUP-1 protein. While we are unclear as to how many NUP-1 molecules contribute to these structures, this, together with data below (Figure 3), suggests that the protein is highly extended (Figure 2D). It is also unclear at this time if NUP-1 is the sole component of the network or if other proteins contribute. Regardless, these data suggest that NUP-1 is a *bona fide* component of the nucleoskeletal network and, moreover, that the location is highly distinct from the punctate staining that we have reported previously for over 20 NPC proteins [23],[36]. We propose referring to NUP-1 as nuclear peripheral protein 1 to help differentiate it from nucleoporins.

To determine if NUP-1 was stably associated with the nuclear periphery, we used fluorescence recovery after photobleaching (FRAP). After bleaching, no significant fluorescence recovery was seen during 150 s (Figure 2B, Movie S5), while fluorescence recovery was observed almost immediately with NLS-tagged GFP (unpublished data). This suggests that NUP-1 is part of a comparatively immobile network at the nuclear periphery in interphase and that rapid exchange of NUP-1 subunits does not occur.

### Architecture of NUP-1 During the Mitotic Cell Cycle

In trypanosomes nuclear mitosis is preceded by division of the kinetoplast, the mitochondrial DNA, while cytokinesis lags behind mitosis by a considerable period. This allows early steps in mitosis to be conveniently detected in mounts of log-phase parasites, and also for post-mitotic nuclei to be analysed within the mother cell prior to cell division. Overall the NUP-1-containing network remained in place at all stages of mitosis (Figure 2A). An extension of the NUP-1 network was also clearly present within the midbody between nuclei in anaphase, which suggests that the NUP-1 network retains intimate contact with the nuclear envelope of the midbody. Note that at late anaphase the midbody becomes depleted of DNA, which has accumulated at the distal poles of the daughter nuclei, so that the midbody is no longer stained significantly with DAPI at this stage.

Despite the maintenance of a clear NUP-1 presence at the nuclear envelope throughout mitosis, significantly less NUP-1 was present in the proximal compared to distal portions of each daughter nucleus (Figure 2C). This rearrangement may contribute to mechanical weakening of the NE to facilitate nuclear fission and strengthening of the distal regions where the spindle is attached. We also found that the overall distance between these NUP-1 puncta increased in mitotic cells compared to interphase, which further suggests remodelling of the network in a cell-cycle-dependent manner (Figure 2D). It is also likely significant that the distance between NUP-1 punctate structures is of the order of 400 nm (Figure 2A and D), suggesting that there is a highly organized assembly of the NUP-1 protein in the trypanosome nucleoskeleton.

We also frequently observed a small spot of NUP-1 between post-mitotic nuclei, apparently unassociated with DNA as detected by DAPI (Figure S1, Movie S4). To determine if this was a genuine extranuclear localization, we compared the location of NUP-1 with NLS-tagged GFP, used to mark the nucleoplasm. This revealed that the NUP-1 spot localized to the residual midbody connection between daughter nuclei at terminal mitotic stages as it also contained GFP (Figure S1D,E). This suggests that a nucleus-derived fragment remains between the daughter nuclei following mitosis, which does not contain significant amounts of DNA; this is presumably a result of the fission mechanism, perhaps analogous to generation of an aerosol when water drops from a faucet. The presence of nucleoplasmic-targeted GFP suggests that this structure, which is only seen in post-mitotic cells and therefore probably rapidly degraded, is unlikely to be stably associated with a cytoplasmic structure (e.g., an MTOC).

### Arrangement of NUP-1 Protein within the Network and Nuclear Targeting Signal

To test whether NUP-1 is distributed throughout the nucleoskeletal network, we stained cells expressing NUP-1-GFP with antibodies against both the C-terminal GFP tag and the repeat region. Given an optical resolution of ∼200 nm, we reasoned that if NUP-1 were predominantly globular, then complete overlap between the two signals would be observed, while if NUP-1 were predominantly elongated, and given a total overall length for the fully extended protein of ∼400 nm, then there would be partial separation of the two signals (Figure 3A). While there was clearly substantial overlap between the spatial distribution of the repeat and C-terminal stains, partial separation was indeed observed (Figure 3B), with the most likely interpretation that NUP-1 is predominantly extended. To confirm the specificity of the co-stain with anti-GFP and anti-repeat antibodies, we also stained cells expressing NUP98-GFP, a nucleoporin, with the NUP-1 anti-repeat antibody (Figure 3B), which demonstrated no cross-reactivity. By contrast, when the GFP epitope was part of a smaller and more compacted FG-repeat-containing nucleoporin, TbNUP89 (Figure 3C), there was clear coincidence between the GFP-stain and that obtained from a cross-reacting antibody to vertebrate NUP107 [36]. These data suggest that at least some NUP-1 polypeptides have an extended conformation, but substantially more data are required to fully understand the architecture of the NUP-1 network.

NUP-1 has a predicted C-terminal nuclear localization signal (residues 3633–3643 of 3647) (http://cubic.bioc.columbia.edu/services/predictNLS/). We tested if this sequence was functional by eliminating residues 3633–3647 by fusing GFP in situ to the *NUP-1* ORF upstream of the putative nuclear localization signal. This resulted in nuclear localization being disrupted, and which was restored by adding back the nuclear localization signal to the *in situ* construct, indicating that the region 3633–3647 is necessary and sufficient for nuclear targeting (Figure S1). A more extensive truncation, deleting the C-terminal domain but adding a nuclear localization signal at the new C-terminus, correctly targeted NUP-1 to the nucleus, and the localization of this truncation appeared indistinguishable from the full-length protein. This suggests that the C-terminal domain is not essential for incorporation of NUP-1 into the nucleoskeletal network.

### NUP-1 Is Necessary for Maintenance of Specific Aspects of the Nuclear Architecture

**Figure 4:**
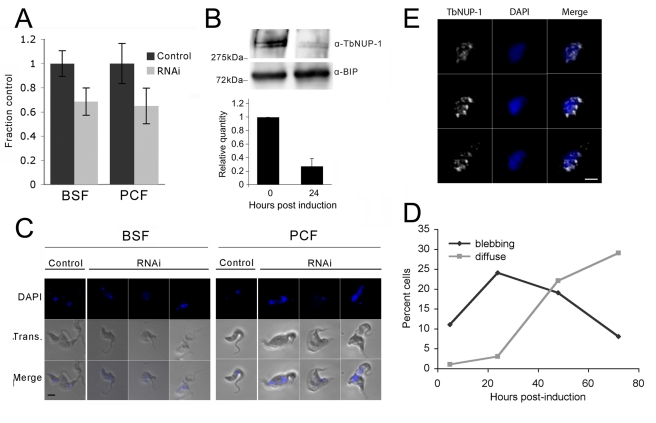
NUP-1 is necessary for cell growth and maintenance of nuclear architecture. NUP-1 RNAi was induced in both BSF and PCF cells. (A, B) NUP-1 mRNA was depleted by ∼30% as measured by qRT-PCR normalized to β-tubulin, corresponding to a ∼75% decrease in NUP-1 protein after 24 h of RNAi induction. Western bands were quantified, normalized to BIP levels, and represented as bar graphs. Error bars are the result of two experimental replicates. (C) IFA using DAPI to visualize DNA (blue). Nuclei of control cells were ovoid with well-defined boundaries, and induced cells exhibited nuclei with diffuse boundaries (i.e., rather than the DAPI nuclear signal being sharply demarcated, the signal gradually decreases with distance from the nuclear centre), and abnormal protrusions (blebs) in both BSF (left panel) and PCF (right panel) cells are seen. Bar: 5 µm. (D) Nuclei with abnormal extensions (blebbing) and with diffuse boundaries were examined over a time course of NUP-1 RNAi induction in BSF cells. 200 cells were scored for each time point. Percentage of nuclei with blebbing or diffuse phenotypes is shown, with the remainder of cells demonstrating a normal phenotype. Initially an increase in cells with bleb nuclei (black) was observed, followed by a decrease in bleb nuclei and an increase in cells with diffuse nuclei (gray). Note that these features are present at less than 1% in an uninduced population. Bar: 2 µm. (E) NUP-1 knockdown BSF cells were probed with an anti-NUP-1 antibody (white). DAPI is used to visualize DNA (blue). Shown are serial sections along the *z*-axis. Bar: 2 µm.

We used RNAi-mediated knockdown to suppress expression of *NUP-1* mRNA in BSF and PCF trypanosomes. 24 h (BSF) or 48 h (PCF) after induction of dsRNA, *NUP-1* mRNA abundance decreased by ∼35%, corresponding to the onset of proliferative defects (Figure 4A, Figure S2A). By 24 h post-induction NUP-1 protein levels in BSF cells were depleted by ∼75% (Figure 4B) at which time gross alterations to the localization of the DNA as stained by DAPI were observed, including nuclear enlargement, abnormal extensions (blebbing), and irregular boundaries (examples in Figure 4C, quantitated in Figure 4D). These resemble the morphological changes observed in numerous laminopathies [8],[9]. We used BSF cells for subsequent analyses except where specified. Residual NUP-1 at 24 h post-induction was collapsed in patches rather than evenly distributed around the nuclear periphery, suggesting compromised organization (Figure 4E).

NUP-1 knockdown led to a decreased proportion of interphase cells (1K1N) and increased proportion of cells entering mitosis/cytokinesis (2K1N, 2K2N) and atypical cells with abnormal nuclei and atypical copy numbers of nuclei or kinetoplasts (Figure S2C “other”). A peak of blebbing structures was followed by a peak of DNA presenting a diffuse boundary (Figure 4D), suggesting that blebbing might allow DNA to spill out of the nuclear remnant. As blebbing was initially observable in mitotic cells, we suggest that NUP-1 depletion results in loss of structural integrity during mitosis and consequent failure to complete mitosis. Interestingly, some distorted nuclei retained the ability to form a spindle as demonstrated by the presence of an intranuclear gamma-tubulin (KMX) rhomboid in a similar proportion of mitotic cells as seen with uninduced cells. This suggests that NUP-1 is unlikely to play a direct role in spindle formation (Figure S2C). Overall, these data indicate a loss of the normal morphology and hence organization of the nucleus upon NUP-1 depletion, which included abnormal midbody organisation in NUP-1 depleted cells that achieved late mitosis. We suggest that failure to complete mitosis is due to rupture of the nuclear envelope (example; left hand procyclic cell Figure 4C).

**Figure 5:**
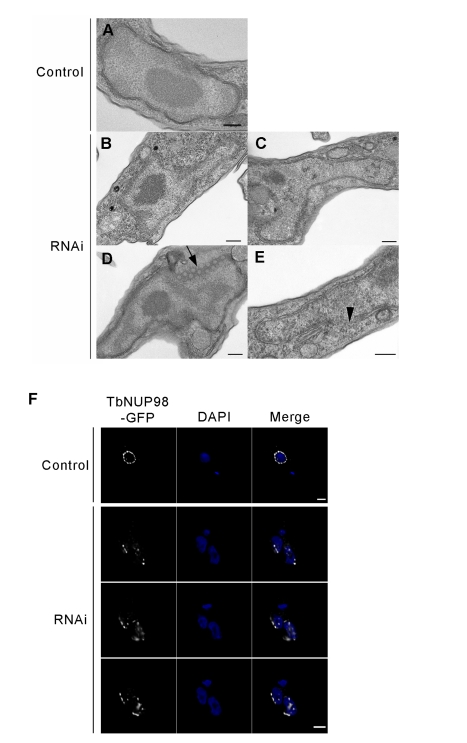
Nuclear morphology and NPC positioning are dependent on NUP-1. (A) Control cell nucleus. (B–C) Nuclear morphology disruption in NUP-1 RNAi cells, with nuclei producing asymmetric extensions and/or invaginations. (D) NPC arrays (black arrow) are visible. (E) Areas of the nuclear envelope appeared ill-defined (black arrowhead). Bar: 200 nm. (F) TbNUP98-GFP (white) was used as a marker of the NPC and in control cells displayed as puncta around the nuclear periphery (top panel). In NUP-1 RNAi cells, NPCs clustered in distinct regions of the nuclear periphery (bottom panels, serial images along the *z*-axis). DAPI was used to visualize DNA (blue). Bar: 2 µm.

By transmission electron microscopy, many knockdown cells had irregular and asymmetric nuclei (Figure 5A–C). Significantly the ER, Golgi, flagellum, and kinetoplast appeared unperturbed, indicating specific nuclear defects (unpublished data). We observed portions of the NE that lost sharp definition, likely due to NE crenelation (multiple small invaginations; 43% of cells, *n* = 28). Such a feature is much less frequently observed in wild type cells (16% of cells, *n* = 25) (Figure 4E, arrowhead). Some 18% of cells (*n* = 35; 0% in wild type, *n* = 25) exhibited quasi-arrays of circular structures that have the same diameter as NPCs (Figure 5D); clustered NPC arrays are also seen with metazoan lamin defects [39],[40]. To verify the roles of NUP-1 in NPC spacing, and also to provide verification that the NPCs had clustered as suggested by EM, we in situ tagged the trypanosome FG-repeat nucleoporin TbNup98 with GFP [36], and monitored the positioning of this protein following NUP-1 depletion. In uninduced cells TbNup98-GFP was observed as regularly spaced puncta surrounding the nucleus (Figure 5F), consistent with our earlier observations and with TbNup98 being a bona fide nucleoporin [36]. In NUP-1 downregulated cells TbNup98-GFP clustered in patches at the nuclear periphery (Figure 5F, Movie S6). We conclude that NUP-1 is required to correctly position and space NPCs at the nuclear envelope, a function performed by lamins in metazoans.

### NUP-1 Is Required for Chromosome Organization

Given the clear role in maintaining nuclear architecture we asked if NUP-1 functions in chromatin organization. *T. brucei* contains 22 chromosomes of ∼1.1–6 Mbp referred to as megabase chromosomes, and several intermediate-sized chromosomes of ∼150–400 kbp. These chromosomes collectively carry the housekeeping genes, *VSG* basic copy genes, and ∼20 subtelomeric *VSG* expression sites [41],[42]. Additionally, there are ∼100 minichromosomes that contain simple sequence repeats and non-transcribed *VSG* genes [43]–[45].

**Figure 6:**
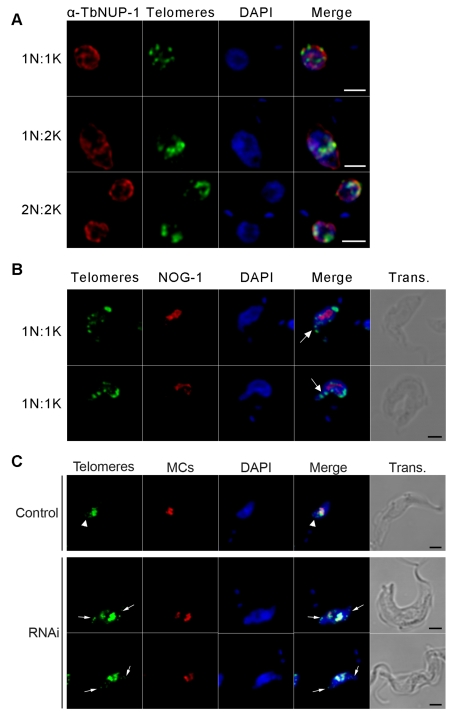
Chromatin organization is dependent on NUP-1. (A) Cells were probed with an anti-NUP-1 antibody (red) and FISH for telomeres (green). DAPI was used to visualize DNA (blue). NUP-1 and telomeres are in close contact throughout the cell cycle. (B) Telomeres (green) were observed in nuclear blebs (white arrows). An anti-NOG-1 antibody (red) was used to visualize the nucleolus and DAPI for DNA (blue). (C) FISH for telomeres (green) and minichromosomes (red). Telomeres marked by only green fluorescence are megabase chromosomes. In control cells, (top panel) megabase chromosomes did not condense as far towards the centre of a dividing nucleus as the minichromosomes (white arrow heads). In NUP-1 depleted cells, megabase chromosomes co-localized with nuclear blebs (white arrows). DAPI was used to visualize DNA (blue). Bar: 2 µm for all panels.

We performed fluorescence in situ hybridization (FISH) with a telomere probe recognizing all chromosomes. We observed NUP-1 partially juxtaposed with telomeres throughout the cell cycle, suggesting some coordination in their movements (Figure 6A). Following NUP-1 RNAi, however, telomeres became clustered and some became located within the nuclear blebs (Figure 6B, arrows). To discriminate between megabase and minichromosomes, we simultaneously used FISH probes specific to megabase and minichromosomal telomeres and a minichromosome-specific probe. In uninduced cells, although most of the telomere and minichromosome FISH signals colocalize, some of the megabase chromosome telomeres (those that hybridized exclusively with the telomere probe) were further from the nuclear centre than minichromosomes (which hybridize with both probes) when aligned for nuclear division, as previously reported (Figure 6C, control) [35],[45]. In NUP-1 knockdown cells, megabase telomeres were the predominant telomere FISH signal in the nuclear blebs, where the minichromosome FISH probe was absent (Figure 6C, arrows); the majority of the telomere FISH signal, however, colocalized with the minichromosome FISH probe in the body of the nucleus (Figure 6C, yellow). Therefore, NUP-1 depletion had a more prominent impact on megabase telomere chromosome positioning than on the minichromosomes.

### NUP-1 Regulates Gene Expression at Telomere-Proximal Regions

As NUP-1 appears to interact with chromatin, based on the location of the NUP-1 protein within a nucleoskeletal network at the nuclear periphery and telomere and NPC positional effects observed by knockdown, we asked if NUP-1 influences telomere-proximal gene transcription. We used several complementary assays to address this issue.

**Figure 7:**
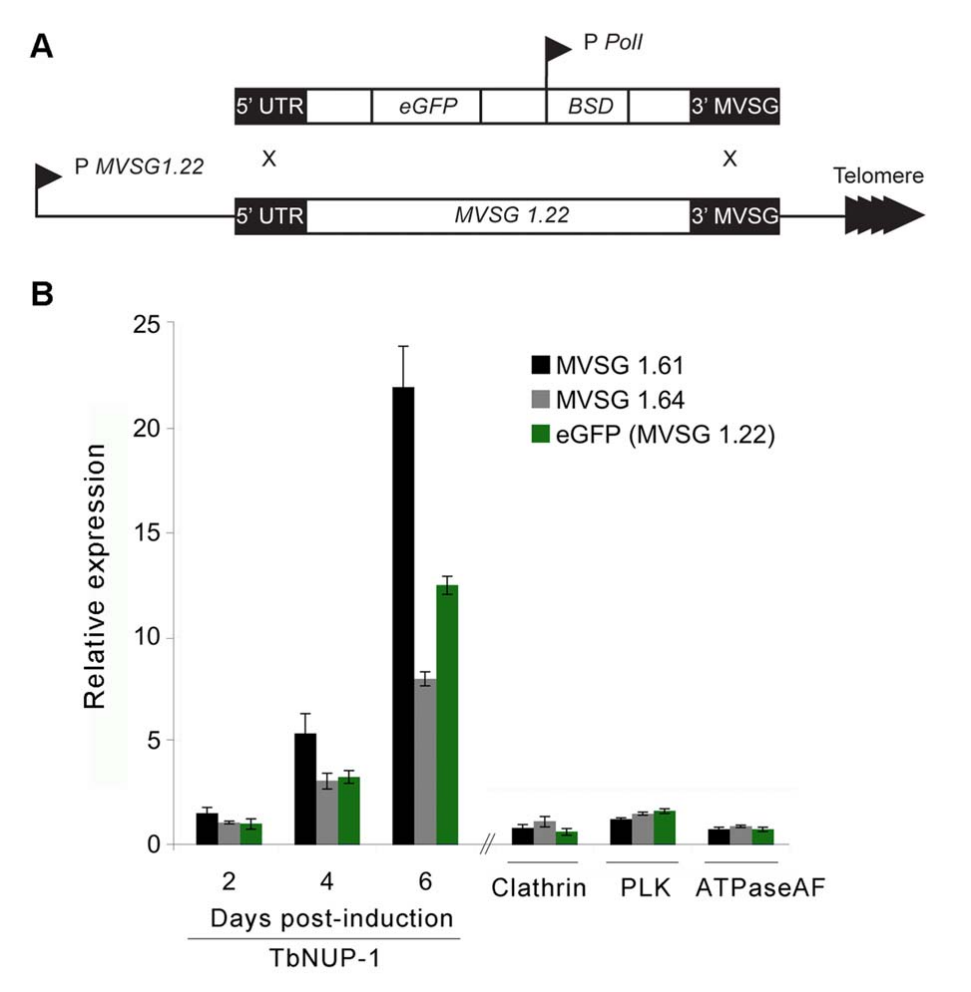
NUP-1 depletion causes misregulation of metacyclic *VSG* (*MVSG*) genes in PCF cells. (A) eGFP reporter modification of the telomeric MVSG 1.22 locus in PCF cells. P, promoter; BSD, blasticidin^R^ gene; Pol I, RNA polymerase l. (B) NUP-1 RNAi induction causes time-dependent increase in *MVSG* and eGFP expression. All values are fold expression compared to uninduced cells determined by qRT-PCR and normalized to β-tubulin. Clathrin, PLK, and ATPaseAF; RNA was isolated after manifestation of the proliferative/morphological phenotype.

Expression of *MVSG* genes, a specific subset of *VSG* genes, is restricted to metacyclic stage *T. brucei* (the life stage present in the tsetse fly salivary gland and injected into a host), and these genes are transcriptionally silent in procyclics [46],[47]. The *MVSG* position, directly upstream of the telomeres, makes them an excellent model to investigate positional effects at subtelomeric sites. Hence, we first induced NUP-1 RNAi in the MVSG 1.22 eGFP PCF reporter cell line (Figure 7A). RNA level derived from two metacyclic *VSG* genes and also the eGFP transgene integrated into an *MVSG* locus was monitored by qRT-PCR. NUP-1 knockdown led to a notably (8- to 22-fold) increased abundance of all three *MVSG* locus mRNAs (Figure 7B). To discriminate between non-specific *MVSG* induction and NUP-1-dependent effects, we individually silenced three unrelated but essential genes: polo-like kinase (PLK) [48], F_0_–F_1_ ATPase associated factor (ATPaseAF) [49], and clathrin [50]. RNAi against each resulted in very severe proliferative and/or morphological defects (Figure S3A) but no significant increase to *MVSG* transcription (Figure 7B), confirming the specificity of the NUP-1 knockdown effect on misregulation of *MVSG* expression.

**Figure 8:**
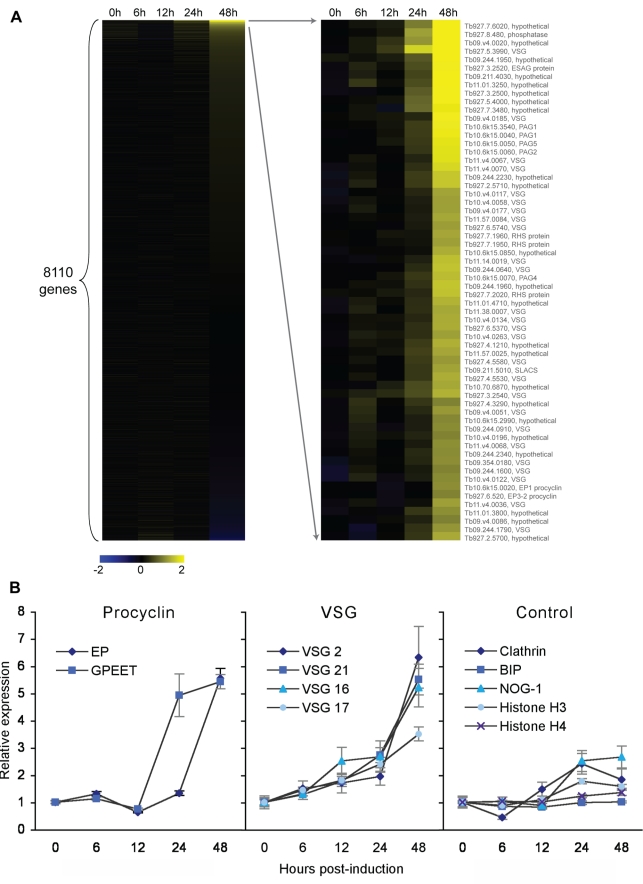
NUP-1 depletion causes increased expression of developmentally regulated genes in BSF cells. (A) Heatmap for microarray following NUP-1 knockdown in BSF cells. Of 8,110 genes, 62 were increased in relative expression greater than 2-fold. Yellow and blue represent upregulation and downregulation, respectively, and black no expression change. Gene accession numbers/annotation are given for the upregulated genes. (B) qRT-PCR quantitation of the expression of *EP* and *GPEET procyclin* genes (left panel), selected ES *VSG* genes (middle panel), and housekeeping genes (right panel) during NUP-1 RNAi. The expression level in control cells was set to one, and *y*-axis is the relative expression compared to control cells, normalized to β-tubulin.

Next we asked if NUP-1 regulates gene expression in BSF *T. brucei* using a microarray to assess global gene expression changes (GEO accession GSE26256) [51]. Of 8,110 genes represented, relative expression levels of 62 genes were upregulated greater than 2-fold, a standard stringent cutoff for significance in microarray experiments (λ>40 cutoff, corresponding to an estimated false discovery rate of 4.93×10^−4^) and demonstrated specific and not global transcriptional changes (Figure 8A, Figure S3B, Table S3). The upregulated gene cohort contained procyclin and VSG genes together with a number of small or repetitive ORFs; significantly no other developmentally regulated or housekeeping ORFs were found. No genes exhibited significant downregulation within these parameters.

Seven upregulated genes were *procyclins* or *procyclin-associated genes* (*p* value = 3.54×10^−12^), residing at two unlinked RNA polymerase I (PolI)–transcribed loci (Figure 8A, Figure S3B, Table S3) [52]. As procyclin is the developmentally regulated protein coat expressed exclusively in PCF cells, derepression in BSF *T. brucei* suggests NUP-1-dependent life-cycle-specific silencing of the procyclin locus [53]. The upregulation of transcription at the procyclin locus was verified by qRT-PCR specific for the two major forms of procyclin, EP and GPEET (Figure 8B). Though GPEET was not identified on the microarray, possibly due to the stringent cutoff, by qRT-PCR, it was also detected as upregulated.

Most significantly, 26 derepressed genes were annotated as *VSG*s (*p* value = 2.03×10^−25^) (Figure 8A, Figure S3B, Table S3). African trypanosomes achieve expression of a single *VSG* gene, and thus antigenic variation, by silencing all but one *VSG* coding sequence present in expression sites and by selective incorporation of a single *VSG* gene within the expression site body [29],[30],[52],[54]. *VSG* genes not located in an expression site are transcriptionally silent. As oligonucleotides specific for expression site *VSG*s were not present on the microarray, the *VSG*s identified by the microarray could correspond to basic copy *VSG*s. However, the *VSG* family is highly homologous and 50% of the *VSG*s identified by the microarray were strongly homologous to one of the 13 (of an estimated 15) sequenced *T. brucei* expression site *VSG*s (unpublished data) [42]. To further investigate if expression site *VSG*s were misregulated by NUP-1 RNAi and if cross-hybridization with expression site *VSG*s could account for the derepressed *VSG*s, we performed qRT-PCR for four expression site *VSG*s. A highly significant increase in *VSG* mRNA was observed (Figure 8B). Amplified products were sequenced and aligned with the most homologous microarray oligonucleotide. Expression site *VSG* sequences from NUP-1 RNAi cells were identical to the published sequences, and large regions of identity were also present between the oligonucleotides and the amplified products (Figure S3C). Though we cannot rule out increased transcriptional activity from basic copy *VSG*s upon NUP-1 knockdown, we do detect mRNA from misregulated expression site *VSG*s and therefore can conclude that NUP-1 participates in maintaining the inactive transcriptional status at the expression sites in BSF *T. brucei*.

### NUP-1 Depletion Derepresses the Entire BSF Expression Site

**Figure 9:**
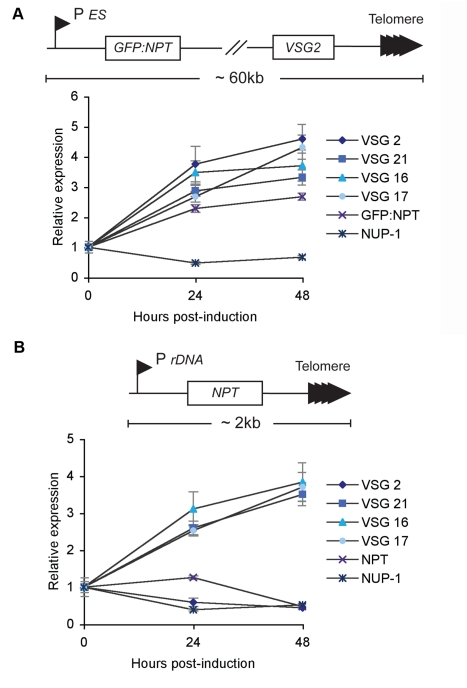
Depletion of NUP-1 derepresses the entire expression site, but not all telomeric genes. (A) Top: NUP-1 RNAi was induced in cells with a *GFP:NPT* reporter downstream of the repressed *VSG 2* expression site promoter. Bottom: Expression of *VSG 2* and the reporter increased as determined by qRT-PCR normalized to Rab11. (B) Top: NUP-1 RNAi was induced in a cell line with *NPT* reporter ∼2 kb upstream of a *de novo* telomere. Bottom: Expression site *VSG* gene expression increased but *NPT* expression did not as determined by qRT-PCR normalized to Rab11. Expression of *VSG 2*, the active *VSG* in this cell line, decreased.

The expression site comprises a PolI promoter driving a polycistronic transcription unit, at the distal end of which resides the *VSG* gene. The *VSG* gene is proximal to the telomere, and in the case of *VSG 2*, the entire locus is ∼60 kb [55]. We asked if misregulation associated with NUP-1 knockdown was restricted to expression site *VSG* transcripts or if the entire expression site was affected. We knocked down NUP-1 in cells containing a *GFP-neomycin phosphotransferase* (*NPT*) reporter close to the *VSG 2* expression site promoter (Figure 9A). Using qRT-PCR the transcriptional levels of the reporter and the *VSG* were found to increase upon NUP-1 knockdown, although changes in protein levels were not detected by Western blot (Figure S4). This suggests a role for NUP-1-mediated control of expression levels across the entire expression site (Figure 9A). To investigate a NUP-1 role in transcriptional control of all telomeric gene sequences, we depleted NUP-1 in cells containing a *NPT* reporter upstream of a *de novo* telomere, under control of a PolI transcribed ribosomal DNA promoter (Figure 9B) [56]. Though *VSG* expression increased, confirming the earlier result, *NPT* mRNA expression decreased during the experiment (Figure 9B). Significantly, NUP-1 knockdown also decreased the expression of the active *VSG* (VSG 2; Figure 9B); the mechanism behind this is presently unclear but may be related to an increased switch frequency (i.e., that a proportion of cells have switched away from expression of VSG 2; see below). As a control for specificity, we also monitored the effect of knockdown of an NPC protein (TbNup98) and analysed the effect on transcriptional misregulation at subtelomeric regions and also the effect on telomere positioning throughout the cell cycle. No major effect was observed for any of these assays (Figure S5), confirming that the effects seen with NUP-1 knockdown are specific and not the result of generic nuclear insult. Therefore, NUP-1 depletion does not induce an increase in transcriptional levels of all telomeric transcripts and demonstrates a specific role for NUP-1 in the transcriptional regulation of developmentally regulated expression site–associated sequences.

### VSG Expression Site Regulation Is Affected by NUP-1 Knockdown

**Figure 10:**
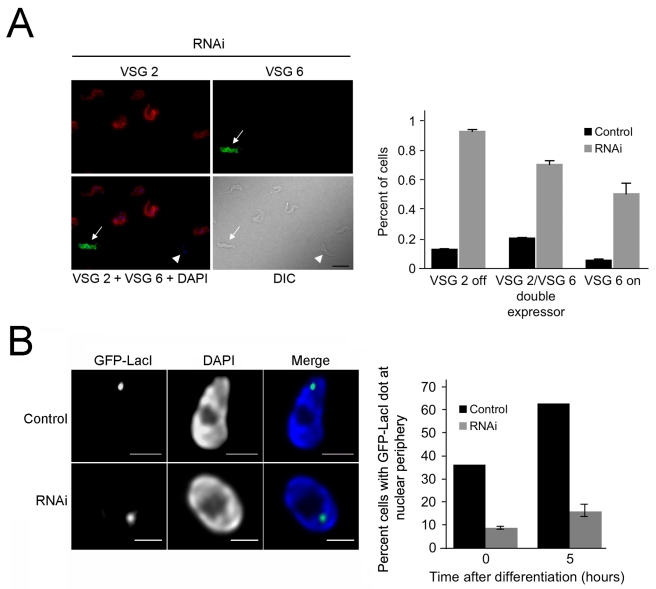
NUP-1 knockdown leads to increased VSG switching and represses differentiation-induced expression site repositioning. (A) Top: Cells were probed with antibodies against the active *VSG 2* (red) and an alternate *VSG 6* (green) following 96 h of NUP-1 RNAi induction. Cells switched from expressing *VSG 2* to *VSG 6* (white arrow), expressed neither *VSG 2* nor *VSG 6* (white arrowhead), or expressed both (not shown). Bar: 10 µm. Bottom: Quantitation of increased *VSG* switching in NUP-1 knockdown cells. Graph represents average and standard deviation of three independent NUP-1 RNAi lines (*n* = 1,000 cells). (B) NUP-1 depletion represses differentiation-induced repositioning of the active expression site to the nuclear periphery. Left: Representative images of maximum intensity projections of 3-D data sets of the GFP-LacI tagged active expression site (green) in wild type cells or NUP-1 RNAi cells (induced for 24 h) after induction of differentiation. Bar: 1 µm. Right: Statistical analysis of the expression site position. The active expression site rapidly repositions to the nuclear periphery in wild type cells. Repositioning is significantly inhibited in NUP-1 RNAi cells. Graph represents the average of three replicate experiments.

The frequency of VSG switching in the Lister 427 cell line is naturally extremely low, with ∼0.1% of cells having switched away from the predominant VSG2. Hence, we used immunofluorescence to determine if the transcriptional decrease in the expressed *VSG* and increase in mRNA from the previously silent expression site *VSG*s coincides with protein production and surface antigen switching. NUP-1 depleted cells were analyzed using antibodies against the expressed VSG (VSG2) and an alternate VSG located in a normally silent expression site (VSG 6). A significant, nearly 10-fold increase in the frequency of cells expressing VSG 6 on their surface following NUP-1 depletion was found (Figure 10A). Double positive cells (i.e., cells that expressed both VSG 2 and VSG 6) were also detected at increased frequency, and as expected due to the slow turnover of the original VSG 2 following initiation of expression of VSG 6. We also observed an increased frequency of cells negative for both VSG 2 and VSG 6 suggesting random switching away from VSG 2 to VSGs other than VSG 6 (unpublished data). These data suggest that NUP-1 depletion not only leads to misregulation of the *VSG* at the mRNA level, but that this encompasses an increase in the frequency of antigenic switching.

This increase in antigenic switching, together with a role in telomeric positioning, may suggest that NUP-1 plays a role in regulating expression site positioning at the nuclear periphery and thus might affect the frequency of translocation of inactive ESs into the nuclear interior and subsequent insertion into the expression site body. The expression site VSG promoter is rapidly repositioned to the nuclear periphery upon developmental differentiation from BSF to PCF, a life stage where no VSGs are expressed [57]. To investigate this issue, NUP-1 was knocked down in BSF cells where the active expression site promoter has been tagged with GFP-LacI, as a marker for the expression site body. These cells were then induced to differentiate into PFs. The active VSG-expression site promoter relocated to the nuclear envelope early during differentiation (5 h) in 63% of control cell nuclei, as expected (Figure 10B). Statistical analysis of the GFP-LacI position in cells depleted for NUP-1 indicated that only 16%±3.2% of the nuclei displayed the GFP-LacI spot at the nuclear periphery (Figure 10B). As efficient incorporation of the previously active *VSG* expression site into the peripheral region of the nucleus is disrupted in the NUP-1 knockdown, these data suggest that developmental silencing of the active expression site at the nuclear periphery requires NUP-1 function, consistent with a role for NUP-1 in peripheral chromatin organization.

## Discussion

While structures that resemble the nucleoskeletal metazoan lamina and heterochromatin are recognized in many eukaryotic lineages, the lamin proteins themselves are clearly restricted to the metazoa [7],[15], and no lamina components have been unequivocally identified to date in any non-metazoan organism. This, together with a very clear absence of lamins and a morpholgical lamina from *S. cerevisiae* and other yeasts, suggests that the lamina arose during differentiation of the metazoan lineage [17]. This distinction implies that the mechanisms for organization of peripheral heterochromatin are substantially divergent throughout the Eukaryota and, more significantly, that the mechanisms understood as present in Metazoa are modern and lineage-specific. An alternate model, that the nuclear lamina is an ancient feature of eukaryotes but has been lost in yeasts, is also possible, especially as it is clear that *S. cerevisiae* has experienced other significant secondary losses. Resolving this issue requires the identification and molecular and functional characterization of a nucleoskeletal structure from a non-metazoan lineage. Here we describe the location and function of one such nucleoskeletal structure from trypanosomes, which are members of the Excavata supergroup, and in evolutionary terms are extremely distant from the Metazoa and also undergo a closed mitosis, probably the dominant form of mitosis in an evolutionary context.

### NUP-1 Forms a Network in the Trypanosome Nucleus

NUP-1 is a predominantly coiled-coil protein that localizes to a lattice-like network at the nuclear periphery. As initially characterised, NUP-1 was suggested to be located as puncta at the nuclear envelope and was proposed to be a nucleoporin (hence the name NUP-1) [35]. By contrast, our earlier work suggested that NUP-1 was associated with fibrillar material on the inner nuclear envelope, which was depleted from regions of the envelope subtending the NPC [23]. Here, for the first time, using *in situ* GFP tagging and high-resolution microscopy, we have revealed that NUP-1 is in fact associated with a network, reminiscent of the lamin nucleoskeleton. We find that several aspects of nuclear organization are extremely sensitive to decreased expression of NUP-1, extending to proliferative defects, structural abnormalities, and NPC clustering; all of these are analogous to phenotypes observed when lamin expression or functions are perturbed. Most significantly, NUP-1 organizes trypanosome chromatin and is required for controlling developmentally regulated and telomere-proximal gene expression, as are lamins. NUP-1 knockdown leads to misregulation of multiple expression site *VSG*s, the *procyclin* loci, and *MVSG*s, all developmentally regulated genes. It is possible that NUP-1 interacts even more extensively with chromatin, with subtle changes below our current detection level. NUP-1 and lamins do, however, demonstrate unique functional aspects: first, as trypanosomes undergo closed mitosis, NUP-1 can have no role in post-mitotic nuclear envelope reformation, but may act in mitotic scission, and second, NUP-1 does not, from presently available data, appear to participate in spindle formation. Taken together, these data argue strongly that NUP-1 is a component of a nucleoskeletal cage within the trypanosome nucleus that supports many functions equivalent to the lamin network of metazoan cells.

### NUP-1 Plays a Role in Telomeric Silencing

The role of NUP-1 in silencing developmentally regulated genes may also provide insights into the process of antigenic variation in trypanosomes. Of the changes to gene expression during life cycle progression in trypanosomes, a major feature is the switch between the dominant surface antigens VSG and procyclin. There are over 1,000 VSG coding sequences in the trypanosome genome, of which only ∼20 occur in the subtelomeric expression sites. Monoallelic expression is achieved by only one expression site being active, in a transcriptionally permissive expression site body environment. This level of selective expression requires a tremendous degree of epigenetic control. While the expression site body partially explains how a single expression site is active, other regulatory mechanisms must constrain the inactive expression sites, securing them against both expression site body entry and spontaneous transcription. This control breaks down on disruption of NUP-1 expression. Following NUP-1 knockdown, megabase chromosome telomeres reposition, multiple expression site *VSG*s become active, and the frequency of VSG coat switching increases. Additionally, the active expression site promoter fails to migrate to the nuclear periphery upon differentiation, suggesting a role for NUP-1 in sequestering and silencing inactive expression sites. As NUP-1 also silences *MVSG* genes in PCF cells, it is likely associated with the formation and maintenance of a repressive heterochromatin environment, paralleling lamin functions [58],[59]. Modulations to the NUP-1 network, involving NUP-1 phosphorylation sites for example, may be a mechanism to release sequestered *VSG* genes to initiate a VSG switch, which is clearly under epigenetic control as trypanosomes rapidly, but reversibly, reduce switch frequency in culture [60]. Overall, our work shows that this mechanism contributes to the extreme level of developmental control of VSG and procyclin, where expression between life stages varies by several orders of magnitude [61]. Significantly, misregulation of VSG and procyclin genes on NUP-1 knockdown is not as extreme, being induced only up to ∼10-fold. This likely requires translocation to an RNA PolI-rich nuclear subdomain (i.e., the expression site body and the nucleolus for VSG and procyclin, respectively).

### Evolution of NUP-1, Lamins, and Nuclear Architecture

The identification here of a component of a nucleoskeletal network in a non-metazoan organism indicates that such structures are not lineage restricted. Taken together with the presence of peripheral heterochromatin and fibrous nucleoskeletons being identifiable in taxa from many lineages, this suggests that the mechanisms for epigenetic control and the tight regulation of specific gene cohorts at the nuclear periphery are likely both a general feature of eukaryotes and an ancient one. This is particularly significant as the mechanisms for controlling the expression of individual mRNAs are highly divergent. For example, in contrast to Metazoa, trypanosomes lack promoter control for the vast majority of genes. Hence, epigenetic control by a peripheral nucleoskeleton, which is entirely absent from prokaryotes, may be among the most fundamental and ancient mechanisms for eukaryotic gene regulation. The question arises as to whether the functional commonality between NUP-1 and lamins has arisen via convergent or divergent evolution. At molecular weights of ∼450 kDa and ∼60 kDa, respectively, these proteins are far from obvious orthologues, while significant sequence relationships are restricted to Metazoa for lamins [15] and trypanosomatids for NUP-1. NUP-1 is apparently absent from the free-living kinetoplastid *Bodo saltans*, and the distant relationships between the *Leishmania* and trypanosome NUP-1 orthologues indicates a great deal of evolutionary plasticity even between more closely related species; African trypanosomes are highly tolerant of perfect repeats, which may explain the extreme expansion of the *T. brucei* form. Conservation of NPCs, nuclear envelope, and heterochromatin clearly argues for a common origin for nuclear organization mechanisms, but many NPC components cannot be identified based on sequence between distant taxa [17],[36],[62], suggesting that overall architecture is more important than primary structure.

A convergent evolution model would imply that coiled-coil proteins organizing peripheral chromatin arose independently in the major eukaryotic lineages, and potentially that the last eukaryotic common ancestor lacked such a feature. Indeed, as lamins are absent from yeast, the understandable hypothesis was that this represented the ancestral state [16]. By contrast a divergent evolution model argues that ancestral coiled-coil proteins diverged into lamins, NUP-1, and presumably similar proteins throughout the eukaryotes (e.g., NMP-1 in plants, with secondary losses in specific lineages such as yeasts). This latter scenario is clearly the most parsimonious when taking the present data into consideration and is in agreement with recent large-scale studies of the evolution of coiled-coil proteins and other protein domains [63]–[65]. Taken together we favour the divergent scenario where the nuclear skeleton evolved along with the nuclear envelope and NPC in the earliest eukaryotes; however, we acknowledge that this issue remains far from settled and will require identification of nucleoskeletal components in additional eukaryotic supergroups for further clarification. A recent report indicates that the Metazoan exclusivity of lamins is incorrect, and a clear highly divergent lamin ortholog is present in *Dictyostelium [79]. This finding has profound implications, strongly suggesting that the fungi have lost an ancestral lamin, rather than metazoa acquiring lamins following separation from the common ancestor with fungi.*

Finally, as nucleoskeletal heterochromatin organization appears crucial for silencing developmentally regulated genes, we speculate that the likely presence of such a system in the last eukaryotic common ancestor implies that this organism had a complex multiple-stage life cycle, requiring nucleoskeletal silencing at the nuclear periphery; this may go some way to explaining the surprising cellular complexity of LECA based on multiple reconstructions. Improved proteomic techniques may lead to identification of lamina components in additional eukaryotic lineages and help to clarify the likely structures within an ancestral nuclear skeleton and the fundamental and conserved processes governing heterochromatin remodelling.

## Materials and Methods

### Cell Culture

BSF *Trypanosoma brucei brucei* MITat 1.2 (M221 strain) and PCF *T. b. brucei* MITat 1.2 (Lister 427) or *T. brucei* EATRO 795 (*MVSG* studies) were grown as previously described [66]–[68]. Single marker BSF (SMB) and PTT PCF lines were used for expression of tetracycline-inducible constructs [67]. Expression of plasmid constructs was maintained using antibiotic selection at the following concentrations: G418 and hygromycin B at 1 µg/ml and phleomycin at 0.1 µg/ml for BSF, G418 at 15 µg/ml, hygromycin B at 25 µg/ml, and phleomycin at 5 µg/ml for PCF.

### In Situ Tagging

ORFs were tagged using the pMOTag4G and pMOTag4H vectors [69] as templates. The following primers were used: NUP-1F: ACAAACACAGCGACAGGTACGGCAAGTCATGGACATACGTAGCACAAGGAAAAGGTCTCGTTCAGCCAATGCGGTCTCGGGTACCGGGCCCCCCCTCGAG; NUP-1R: TCTAGGTGCATGTGTAGATGAACTGCACACTTTATGCACTAATAACAGGTTTGAAGTACTTACCTGGCATCTCCTGGCGGCCGCTCTAGAACTAGTGGAT; Tb927.4.2070F: GAGCTGAGAAAGTGTAATGACTTAGTTATAAAGAGACTAGAGGATGAGGTTAAAGCTCTTCGTGAAGAACTGCGTGGAAATGAGGCGGGTACCGGGCCCCCCCTCGAG; Tb927.4.2070R: CCAATAGAAAAAAATGTAAGTAGCAATAATACGTATTTAAAAATGTCAAAATTGTCAGCAACAAAGATGCTTACACGAACAGAAAAAAAAAGATGGCGGCCGCTCTAGAACTAGTGGAT; Tb927.7.3330F: CGCTCTGTTGAGGAAGGTGAAGACGATGAGGACGAGGGCGACGCAACCGGTTGCCCAACAACGCATTTGGGAGGGCCATGGGCGCACCATGGTACCGGGCCCCCCCTCGAG; Tb927.7.3330R: CAAAAATATTCGTTACATTAGACATCATTCATCGACTGTAACCTAGGTAGTGTATGAGATACCGTATCAATTACACACTGAGTGTCATGGCGGCCGCTCTAGAACTAGTGGAT; TB92733180F: TGGGAATGCTTCAGCAAGTGGTGAAAAGAACAATGCTCCACGGAATCCCTTCTCATTTGGTGCCTCTTCTGGGAATGCTGGTACCGGGCCCCCCCTCGAG; TB92733180R: ACTAAAGAAGGGTAGAAAACAAAGAAAACACCAAATAAGGTACCTGACGCAGCGGCAACACCACGTCGACTTGCTGGCGGCCGCTCTAGAACTAGTGGAT.

For truncation in situ tagging, the following primers were used: NUP-1noNLSF: GGTGAGCTTGTCCGTTGAGTCATCACATCATTCCAGAATCACTGAACAAACACAGCGACAGGTACGGCAAGTCATGGACGGTACCGGGCCCCCCCTCGAG; NUP-1noNLSR: GTTTGAAGTACTTACCTGGCATCTCCTCACGAGACCGCATTGGCTGAACGAGACCTTTTCCTTGTGCTACGTATTGGCGGCCGCTCTAGAACTAGTGGAT; TbNUP1NrepeatsTagF: CCGTACAGCAAAGGAGAAGCTGGAGAGGAGTGTTGAGGAAATATCTTTTTTAAAAGATGAAGTTTTGGTTAGTAATCGTATACGTAGCACAAGGAAAAGGTCTCGTTCAGCCGGTACCGGGCCCCCCCTCGAG; TbNUP1NrepeatsTagR: TCAACATCTGCACCAACAGCACCATCACTATCCCCCACTTTACCATTCAAAGAAGAAACACTATCCACAAGCAATGGCGGCCGCTCTAGAACTAGTGGAT; TbNUP1noNLSplusNLSTagF: GGTGAGCTTGTCCGTTGAGTCATCACATCATTCCAGAATCACTGAACAAACACAGCGACAGGTACGGCAAGTCATGGACATACGTAGCACAAGGAAAAGGTCTCGTTCAGCCGGTACCGGGCCCCCCCTCGAG.

Linear PCR products were purified by ethanol precipitation. Electroporation was performed with 10–25 µg of DNA using a Bio-Rad Gene Pulser II (1.5 kV and 25 µF). Positive clones were assayed for correct insertion and expression using PCR and/or Western blotting.

### Immunofluorescence Analysis

For microscopy, cells were fixed with 3% paraformaldehyde (v/v) for 1 h on ice (PCF) or 15 min at room temperature (BSF) and allowed to settle onto poly-L-lysine coated slides (VWR International) at room temperature. For permeablization, cells were incubated with 0.1% Triton X-100 for 10 min in PBS. Slides were blocked in 20% FBS (Sigma) in PBS for 1 h. Cells were incubated with primary antibody in 20% FBS/PBS for 1 h followed by three 5-min washes in PBS. Cells were incubated with secondary antibody in 20% FBS/PBS followed by three 5-min washes in PBS. Slides were mounted with Vectashield mounting medium plus DAPI (Vectashield Laboratories). Antibodies were used at the following concentrations: rabbit anti-GFP 1∶3,000, goat anti-rabbit IgG Alexa Fluor 488 (Molecular Probes) 1∶1,000, mouse anti-HA (Santa Cruz Biotechnology Inc.) 1∶1,000, goat anti-mouse IgG Alexa Fluor 568 (Molecular Probes) 1∶1,000, polyclonal rabbit anti-NUP-1 (produced by Covalab against the NUP-1 peptide NH_2_-CLNAAGVRVRTSQSDKD-COOH) 1∶750, and rabbit anti-TbNog1 629L (gift from M. Parsons) 1∶700. VSG antibodies and visualization were performed as previously described [70]. For combination immunofluorescence and FISH, samples were processed for immunofluorescence as above and post-fixed in 3% paraformaldehyde for 1 h and then processed for FISH as described below.

### Imaging

Confocal microscopy images were acquired with a Leica TCS-NT confocal microscope with a 63×/1.4 or 100×/1.4 numerical aperture objective. Images were processed with Huygens deconvolution software (Scientific Volume Imaging) and Adobe Photoshop (Adobe Systems Inc.). Quantitation was on raw images. Fluorescence images were acquired using a Nikon Eclipse E600 epifluorescence microscope and a Hamamatsu ORCA charge-coupled device camera. Images were captured using Metamorph software (Universal Imaging Corp.) and raw images processed using Adobe Photoshop.

### Western Blotting

10^7^ cells per lane were resolved on 4%–12% SDS–polyacrylamide gels (Invitrogen). Proteins were transferred to polyvinylidene fluoride membranes (Millipore). Detection of HRP-conjugated secondary antibodies was by chemiluminescence with luminol (Sigma). Polyclonal rabbit anti-TbBiP serum (a gift from J. D. Bangs) was used at 1∶5,000, polyclonal mouse anti-NUP-1 (a gift from K. Ersfeld) at 1∶10,000, mouse anti-VSG 2 (a gift from G.A.M. Cross) at 1∶20,000, rabbit anti-NPT (Fitzgerald Industries International, Inc.) at 1∶5,000, and polyclonal anti-TbRab11 was used at 1∶2,000. Incubations with the appropriate commercial secondary anti-IgG horseradish peroxidase (HRP) conjugates (Sigma) were performed at 1∶10,000 in TBST for 45 min. Image J (National Institutes of Health) was used to quantify band intensity.

### Quantitative RT-PCR and DNA Sequencing

RNA was purified using the RNeasy mini kit (Qiagen) according to the manufacturer's instructions. RNA concentration was quantified using a ND-1000 spectrophotometer and Nanodrop software (Nanodrop Technologies). cDNA was produced using Superscript III Reverse Transcriptase (Invitrogen) according to the manufacturer's instructions. qRT-PCR using cDNA templates was performed with iQ-SYBRGreen Supermix and MiniOpticon Real-Time PCR Detection System (Bio-Rad) and quantified with Opticon3 software (Bio-Rad). β-Tubulin, which has stable mRNA levels throughout the *T. brucei* cell and life cycles, was used for normalization [71]. The following primers were used for qRT-PCR: 3330qRTF: GGGTGTTTTCGTTGATGAGGTCT; 3330qRTR: ACTGCAGCGAAGACAAAGAAGAG; 2070qRTF: GGTTCAGGAGGAGACGATGAAGT; 2070qRTR: AGCTTTAACCTCATCCTCTAGTCTCT; Nup1qRTF: CGAGGAGGAGGTTGGAGGAG; Nup1qRTR: GCTGGCACTCCTTCTGCAATTT; TbBetaTubulinF: CAAGATGGCTGTCACCTTCA; TbBetaTubulinR: GCCAGTGTACCAGTGCAAGA; VSG2F: CCAAGTTAACGACTATACTTGCCTATT; VSG2R: CAAGTAGCAAGGAAAATTTTAAAAGG; VSG21F: CGGATGCTCAAATCTATTACACAG; VSG21R: GTCAGAATTCTTAGAATGCAGCC; VSG16F: AGTCGTAGCACTTTTGATTCAGG; VSG16R: TTATGCTAAAAACAAAACCGCA; VSG17F: CACCAACACAGCAGAACGAA; VSG17R: TTATGCTAGAATCAAAAATGCAAGC; EPProcyclinQF: AAGGACCAGAAGACAAGGGTC; GPEETProcyclinQF: ATGGCACCTCGTTCCCTT; ProcyclinQR: TTAGAATGCGGCAACGAGA; BIPQF: GGTGAGCGCTATGGACAAGT; BIPQR: GTCCTCGTCCTCGAACTCTG; NOG1QF: GCTCACTTAGCGTAAACCGC; NOG1QR: GTATGGCACGATCACCCTCT; HistoneH3QF: GACCTGCTGCTACAAAAGGC; HistoneH3QR: AAGCAGCGACACAATGTACG; HistoneH4QF: TATCTACGACGAGGTGCGTG; and HistoneH4QR: AACCGTACAGAATCTTGCCG.

For sequencing qRT-PCR products, the following primers were used: VSG2sequence: TTTGAAGTTTTAACCCAGAAGCA; VSG21sequence: AAAAAAGAAAAACCAAGCAAGTCC; VSG16sequence: AGAAATGTCCAGCGAGCC; and VSG17sequence: AAGAACAGGAAACTACAAAGGTAGACG.

### Electron Microscopy

Cells were fixed in 2% glutaraldehyde (v/v), resuspended in 0.1 M PIPES, and processed for electron microscopy by the University of Cambridge multi-imaging centre. Sections were viewed on a Philips CM100 electron microscope (FEI-Philips) operated at 80 kV.

### RNA Interference

PCR products were cloned into either the p2T7^TABlue^ plasmid (BSF) [72] or the p2T7-177 plasmid (PCF) [73]. RNAi was induced in log-phase cells by the addition of 1 µg/ml tetracycline. The following primers were designed using RNAit [74]: NUP-1RNAiF: ATCGAAACGTGAGGGTGAAC; NUP-1RNAiR: ACCCTTGTCTTGGCATATCG; Tb927.7.3330RNAiF: CCACAGAACACACCGAAATG; Tb927.7.3330RNAiR: CCTTCTCGTCCAACTCAAGC; Tb927.4.2070RNAiF: TGATCCATTCCCTTGAGGAG; Tb927.4.2070RNAiR: GGGGAGGTGTGTGTCACTCT; NUP-1177RNAiF: GCTACTCGAGATCGAAACGTGAGGGTGAAC; and NUP-1177RNAiR: CGTAGGATCCACCCTTGTCTTGGCATATCG.

### FISH

The telomere PNA FISH kit (DAKO) was used according to the manufacturer's instructions. For combined FISH with the Telomere PNA kit and the Digoxigenin labelled 177 base pair fragment that binds to minichromosomes, cells were pre-treated according to the kit manufacturer's instructions and then prehybridized in hybridization buffer (50% formamide, 2× SSC, 10% dextran sulfate, 50 mM sodium phosphate pH 7) for 45 min. Probe (10 µl kit probe, 5 µl hybridization buffer+minichromosome probe) was added and cells were denatured for 5 min at 88°C. Slides were incubated overnight at 37°C. Slides were washed in 50% formamide/2× SSC for 30 min at 37°C, 2× SSC for 10 min at 50°C, 0.2× SSC for 60 min at 50°C, and 4× SSC for 10 min at room temperature. A rhodamine-conjugated anti-digoxigenin antibody (Fab fragments, Roche) was added at 1∶200 in BMEB (100 mM maleic acid, 150 mM sodium chloride, pH 7.5, 1% blocking reagent; Roche). Slides were washed in 1× TBS+0.05% Tween 20 and imaged as described.

### Bioinformatics

We searched for NUP-1 homologues in representative taxa of major eukaryotic supergroups using BLAST. *Homo sapiens* data were obtained from NCBI (www.ncbi.nlm.nih.gov). *Saccharomyces cerevisiae* was at the Saccharomyces genome database (http://www.yeastgenome.org/). *Drosophila melanogaster* data were at FlyBase (www.flybase.org). *Caenorhabditis elegans* was obtained from WormBase (www.wormbase.org). *Chlamydomonas reinhardtii*, *Ostreococcus tauri*, *Thalassiosira pseudonana*, *Phytophthora ramorum*, and *Naegleria gruberi* data were obtained from the Joint Genome Initiative (genome.jgi-psf.org). *Cryptococcus neoformans*, *Theileria parva*, *Tetrahymena thermophila*, and *Trichomonas vaginalis* data were from TIGR (www.tigr.org). *Dictyostelium discoideum*, *Entamoeba histolytica*, *Plasmodium falciparum*, *Trypanosoma brucei*, *Trypanosoma congolense*, *Trypanosoma brucei gambiense*, *Trypanosoma vivax*, *Trypanosoma cruzi*, *Leishmania major*, *Leishmania infantum*, *Leishmania braziliensis*, and *Bodo saltans* data were obtained from geneDB (www.genedb.org). More complete *Trypanosoma congolense* and *Trypanosoma vivax* sequences were a generous contribution from Andrew Jackson at the Wellcome Trust Sanger Institute. *Toxoplasma gondii* data were from ToxoDB (www.toxodb.org), *Cryptosporidium parvum* data from CryptoDB (www.cryptodb.org), and *Cyanidioschyzon merolae* data were retrieved from the *C. merolae* genome BLAST server (merolae.biol.s.u-tokyo.ac.jp). *Euglena gracilis* data were obtained from the *Euglena gracilis* genome project (http://web.me.com/mfield/Euglena_gracilis/E._gracilis.html).

### Microarrays

Labelled cDNA probes were created from 2 µg of total RNA using the SuperScript indirect cDNA labelling kit. Unincorporated nucleotides were removed by QuickClean resin, and the resulting cDNA was ethanol precipitated overnight at −20°C. Control and experimental samples were labelled with Alexa Fluor 555 and 647, respectively, and purified using the cDNA labelling purification module. Equal amounts of labelled control and experimental samples were combined. Expression microarray was performed with dye-swaps and experimental and technical replicates as described previously [75]. The log_2_ of the average mRNA abundance ratios are reported. Differentially expressed genes were identified by maximum-likelihood analysis (λ>40) [76].

### FRAP

Cells in log phase growth were trapped between a slide and coverslip at 27°C for no longer than 30 min. FRAP experiments were performed on a Zeiss inverted microscope (Axiovert 200) with a 63×/1.4 numerical aperture objective and spinning disk confocal scanning unit (CSU22, Yokogawa). Time-lapse sequences were acquired using Volocity software with an EMCCD camera (C-9100, Hamamatsu) operating in streaming mode. Representative movies are presented. Fluorescence intensity was measured in the region of interest using ImageJ. For the experiments on cells expressing NUP-1-GFP, 28 image sequences were acquired in four independent FRAP experiments. Movies were taken at either maximum speed of acquisition (no time-lapse, 0.221 s between frames, 311 time points) or a time-lapse of 1.98 s between frames (70–140 time points) with an exposure time of 200 ms/frame (binning 1×1 pixels). For the experiments on cells expressing NLS-GFP, 30 image sequences were acquired in five independent FRAP experiments. Movies were taken at maximum speed of acquisition (no time-lapse, 0.068 s between frames, 588–625 time points) with an exposure time of 50 ms/frame (binning 1×1 pixels). To normalize data, background fluorescence was subtracted and overall photobleaching over time normalized using another equivalent source of GFP (a similar nuclear region of a cell that had not been bleached).

### *MVSG* Reporter Cell Line RNA Interference

RNAi line EATRO 795 2913 MVSG1.22->EGFP was obtained by consecutive transfection of EATRO795 PCF cells with pLew13, pLew29, and with a cassette replacing MVSG 1.22 with eGFP. Full details will be published elsewhere. PLK, ATPaseAF, clathrin, and NUP-1 were depleted by RNAi. Three independent RNAi lines were examined for each gene. To generate PLK:RNAi (Tb927.7.6310) and F_0_–F_1_ ATPaseAF:RNAi (Tb10.70.7760) lines, the following primers were used to amplify cDNA fragments: PLKBamHI: GATAGGATCCTTCCCACTGTTTGGGTGACG; PLKXhoI: GAATCTCGAGCAAGCCGTGCAGCAATTTCTCTAG; Tb7760BamHI: GATCGGATCCGAAGCTCAGGACC; and Tb7760XhoI: GATACTCGAGGCAGAAACGCATC.

The corresponding PCR fragments were cloned into the BamHI/XhoI sites of the pZJM plasmid [77] and transfected to the EATRO 795 RNAi line. Clones were selected in the presence of G418 (5 µg/ml), hygromycin B (5 µg/ml), blasticidin S (5 µg/ml), and phleomycin (1.75 µg/ml). Clathrin and NUP-1 RNAi lines were selected in the same way, but DNA fragments were cloned into the p2T7-177 plasmid. For growth rates, *T. brucei* cultures were diluted to ∼7×10^5^ cells/ml and cell densities of parasite cultures evaluated daily. RNAi induced cultures were grown in the presence of tetracycline (2 µg/ml). Cell cultures were diluted to the same density (6–7×10^5^ cells/ml) when cultures reached 1–1.2×10^7^ cells/ml. For quantitative RT-PCR, samples were collected at different time points after TET induction dependent on RNAi line response. Quantitative PCR was carried out on cDNA templates using Power SYBR Green PCR Master Mix (Applied Biosystem, Invitrogen). The following primers were used for qRT-PCR: 161QF: GGCGGTTTGTCTTTGTTTTTG; 161QR: TGACTCCTCTTTGTTGTCGTCTTC; 164QF: CACAGACCTGCAGATGCACTTTAT; 164QR: TGCCTTTATCTTTGCTAAATTTGCT; GFPQF: CTGCTGCCCGACAACCA; and GFPQR: TGTGATCGCGCTTCTCGTT.

### ES and telomeric Reporter Lines

*NUP-1* or TbNup98 RNAi fragments were cloned into the pRPa^iSL^ stem loop RNAi plasmid [77] following amplification with 5iNUP (5′-GATCGGGCCCGGTACCATCGAAACGTGAGGGTGAAC) and 3iNUP (5′-GATCTCTAGAGGATCCACCCTTGTCTTGGCATATCG) or 3iNup98 (5′-GATCTCTAGA GGATCCATAACCGTACGCCTTTGTGC) and 5iNup98 (5′-GATCGGGCCCGGG TGGAGCGGCGTAGTAGAAGT) and digestion with *Kpn*I/*Bam*HI or *Xba*I/*Bam*HI (sense fragment) and *Bsp*120I/*Xba*I or *Bsp1*20I/*Xma*I (antisense fragment). Plasmid was linearized with *Asc*I and transfected into 2T1 strain [78] modified with a sub-telomeric *NPT* reporter 2 kb upstream of a *de novo* telomere (2T1.R2; [56]) or a *GFP:NPT* reporter downstream of the repressed *VSG2* expression site promoter (2T1.*GFP:NPT*; [55]). Three independent RNAi lines were examined for each experiment. RNA was prepared using a Qiagen RNeasy kit following induction of RNAi (1 µg ml^−1^ tetracycline) for 24 and 48 h. The following primers were used for qRT-PCR: NPTQF: TCTGGATTCATCGACTGTGG; NPTQR: GCGATACCGTAAAGCACGAG; RAB11QF: ATCGGCGTGGAGTTTATGAC; and RAB11QR: GTGGTAAATCGAACGGGAGA.

### Active ES Positional Analysis

NUP-1 RNAi was induced for 24 h in a cell line with a GFP-tagged active *VSG* ES promoter. Cells were differentiated and analyzed as in [56].

## Supporting Information

Figure S1 NUP-1 repeat structure, nuclear localization signal, and NUP-1 post-mitotic remnant. (A) Top: A single NUP-1 repeat from selected trypanosomatid species. Black boxes are identical residues and gray boxes similar in at least 50% of the included sequences. Lower: All of the repeats from *T. brucei* are identical with the exception of three conservative substitutions highlighted in red. (B) *NUP-1* mRNA is present at similar levels in both BSF and PCF stages of *T. brucei* as measured by qRT-PCR and normalized to β-tubulin. (C) Wild type NUP-1-GFP (white) localizes to the nucleus (top panel). The nuclear localization is largely disrupted in cells expressing truncated NUP-1-GFP lacking the C-terminal 15 amino acids, which includes a predicted 11 amino acid nuclear localization signal (second panel). When the putative nuclear localization signal is added back onto the in situ tagging construct, nuclear localization is restored (third panel). When the C-terminus of NUP-1 is removed by a nuclear localization signal-containing tagging construct, NUP-1 still localizes to the nuclear periphery, suggesting that the C-terminal domain is not required for targeting (fourth panel). DAPI is used to visualize DNA (blue). Bar: 2 µm. (D) An additional localization was observed for NUP-1-GFP (white) between nuclei in late mitosis (anaphase) (white arrows). DAPI is used to visualize DNA (blue). Bar: 2 µm. (E) Cells expressing NLS-tagged GFP (green) and NUP-1-HA (white) were examined. It was found that NUP-1 localized to a slender connection between daughter nuclei in cells with incompletely segregated nuclei, possibly corresponding to a nuclear remnant remaining between daughter nuclei following mitosis derived from the midbody. DAPI is used to visualize DNA (blue). Bar: 2 µm.
(TIF)

Figure S2 NUP-1 knockdown causes proliferative and NUP-1 lattice defects but does not affect spindle formation. (A) NUP-1 knockdown resulted in a significant growth defect compared to the control population in both BSF and PCF cells. (B) Cell cycle analysis of 200 BSF cells from control and RNAi populations 24 h after RNAi induction revealed a decrease in interphase cells (1N∶1K) and an increase in all other cell types observed. “Other” cells included those with nuclear and kinetoplast arrangements other than those scored in the remaining categories as well as nuclei that had abnormal protrusions and diffuse borders.
(PDF)

Figure S3 Control RNAi disrupts PCF proliferation and BSF NUP-1 RNAi increased expression of developmentally regulated genes. (A) RNAi induction of clathrin, polo-like kinase (PLK), and F_0_-F_1_ ATPase-associated factor (ATPaseAF) (right panel) all caused the expected growth defects in the reporter cell line. −, uninduced; +, induced. (B) Kinetics of procyclin and VSG gene expression changes in response to NUP-1 RNAi. Expression changes are on a logarithmic scale and relative to the expression level prior to RNAi induction and are derived from the array data shown in Figure 8. (C) Expression site VSG PCR products from qRT-PCR were sequenced (VSGXsequencing) and compared to the published bloodstream expression site VSG sequences (VSGX) and the array oligonucleotide from the 62 showing induction of expression most closely similar to the bloodstream expression site VSG (TbXoligo). Black boxes denote 100% sequence conservation.
(PDF)

Figure S4 Protein levels from NUP-1 RNAi derepressed expression sites are not detectable by Western blot. NUP-1 depletion does not lead to protein levels from repressed sub-telomeric loci that are detectable by Western blot. The *VSG 2* expression site and *de novo* telomere silencing cell lines (see Figure 9) were induced for 48 h. Expression of GFP:NPT or VSG 2 from the silenced BES1 is not detectable following RNAi against NUP-1; the parent cell line, where this expression site is active (“Active”), is shown for comparison. Similarly, no increase in NPT protein production was seen following NUP-1 RNAi when the reporter was located ∼2 kb from a *de novo* telomere; this reporter can be derepressed, as shown by the SIR2rp1 and sir2rp1 cell lines included. The Rab11 blot and the Coomassie stained gel serve as loading controls. NPT, neomycin phosphotransferase.
(PDF)

Figure S5 Depletion of Nup98 does not result in telomere mispositioning or misregulation of ES associated sub-telomeric genes. (A) Top panel: RNAi against TbNup98 was induced in cells containing a GFP::NPT reporter downstream of the repressed VSG2 ES promoter. Bottom panel: Expression of ES VSG genes and GFP::NPT reporter do not increase following depletion of TbNup98 as determined by qRT-PCR, normalised to Rab11. Note the difference between the behaviour here and in Figure 9 for NUP-1 knockdown. (B) Top panel: TbNup98 RNAi was induced in cells that contain an NPT reporter ∼2 kb upstream of a *de novo* telomere. Knockdown of TbNup98 resulted in a decrease in the expression of ES VSGs and the NPT reporter as determined by qRT-PCR, again distinct from the effect of NUP-1 RNAi. Knockdown of TbNup98 was validated by monitoring cell proliferation—doubling time was decreased by ∼50% at 24 h. (C) Cells were probed with anti-NUP-1 repeat region antibody (green) and FISH for telomeres (red). DAPI was used to visualise DNA (blue). The distribution of NUP-1 was unaffected by RNAi mediated knockdown of TbNup98. (D) Top panels: FISH images of telomeres in cells knocked down for TbNUP98. Following RNAi, the distribution of telomeres (white) was not substantially altered in interphase or mitotic cells. DAPI was used to visualise the DNA. Bars: 2 µm. Lower panels: Quantitative analysis of telomere positions comparing TbNup98 RNAi cells and uninduced control cells (+ and − Tet, respectively). Data showing the average and standard deviation derived from three independent TbNup98 RNAi cell lines are shown (*n* = 150 for uninduced and *n* = 150 for induced). There is no significant alteration to the telomere positioning or the migration of telomeres during cell division.
(TIF)

Movie S1 NUP-1 forms a network-like structure around the periphery of the nucleus. PCF cells expressing NUP-1-GFP (white) were imaged by confocal microscopy. Shown is an image series along the *z*-axis of a cell at interphase. DAPI is used to visualize DNA (blue).
(MOV)

Movie S2 NUP-1 forms a network-like structure around the periphery of the nucleus. PCF cells expressing NUP-1-GFP (white) were imaged by confocal microscopy. Shown is an image series along the *z*-axis of a cell during nuclear division. DAPI is used to visualize DNA (blue).
(MOV)

Movie S3 NUP-1 forms a network-like structure around the periphery of the nucleus. PCF cells expressing NUP-1-GFP (white) were imaged by confocal microscopy. Shown is an image series along the *z*-axis of a cell during very late nuclear division. DAPI is used to visualize DNA (blue).
(MOV)

Movie S4 NUP-1 forms a network-like structure around the periphery of the nucleus. BSF cells probed with an anti-NUP-1 antibody (white) were imaged by confocal microscopy. Shown is an image series along the *z*-axis of interphase cells. DAPI is used to visualize DNA (blue).
(MOV)

Movie S5 The NUP-1 network around the periphery of the nucleus is stable. Fluorescence recovery after photobleaching (FRAP) was performed on cells expressing NUP-1-GFP (white). Shown is a time-lapse of 1.98 s between frames with an exposure time of 200 ms/frame (binning 1×1 pixels).
(MOV)

Movie S6 Nuclear pore complex positioning is dependent on NUP-1 expression. TbNup98-GFP (white) was used as a marker of the nuclear pore complex. In NUP-1 RNAi cells, the nuclear pore complexes cluster in distinct regions of the nuclear periphery. Shown are serial confocal images along the *z*-axis. DAPI is used to visualize DNA (blue).
(MOV)

Table S1 NUP-1 orthologues in trypanosomatids. Accession numbers of sequences with homology to NUP-1. In species indicated by multiple accession numbers, the NUP-1ORF appears to have been split into multiple parts during genome annotation and assembly.
(PDF)

Table S2 Percent identity/similarity for the alignment of full-length NUP-1 with other trypanosomatid NUP-1 sequences. Percent identity is shown in the lower portion of the table and percent similarity in the upper portion. NUP-1 is most similar to the sequence from *T. b. gambiense*, which is essentially a subspecies of *T. brucei*. Homology is decreased amongst other African trypanosomes and lower still for the South American trypanosome *T. cruzi*. There is very low identity/similarity between NUP-1 and putative orthologues in *Leishmania*. However, the NUP-1 gene remains syntenic in these species.
(PDF)

Table S3 Genes with greater than 2-fold induction of expression following NUP-1 RNAi. 26 VSGs and 23 hypothetical genes accounted for the majority of the 62 upregulated genes. The expression of seven genes from the procyclin genomic loci was also induced. One sequence was annotated as an expression site associated gene (ESAG), and four were retrotransposon-associated sequences. The remaining gene is annotated as a putative phosphatidic acid phosphatase. The full data set is available at array express with the accession GEO26256.
(XLS)

## Abbreviations

BSF: bloodstream form
ES: expression site
FISH: fluorescence in situ hybridization
NE: nuclear envelope
NPC: nuclear pore complex
NPT: neomycin phosphotransferase
PCF: procyclic form
PLK: polo-like kinase
VSG: variant surface glycoprotein
