# Supplementary figures and images for: NUP-1 Is a Large Coiled-Coil Nucleoskeletal Protein in Trypanosomes with Lamin-Like Functions
Source: PLoS Biol. 2012 Mar 27;10(3):e1001287. doi: 10.1371/journal.pbio.1001287 (PMC3313915; doi:10.1371/journal.pbio.1001287)

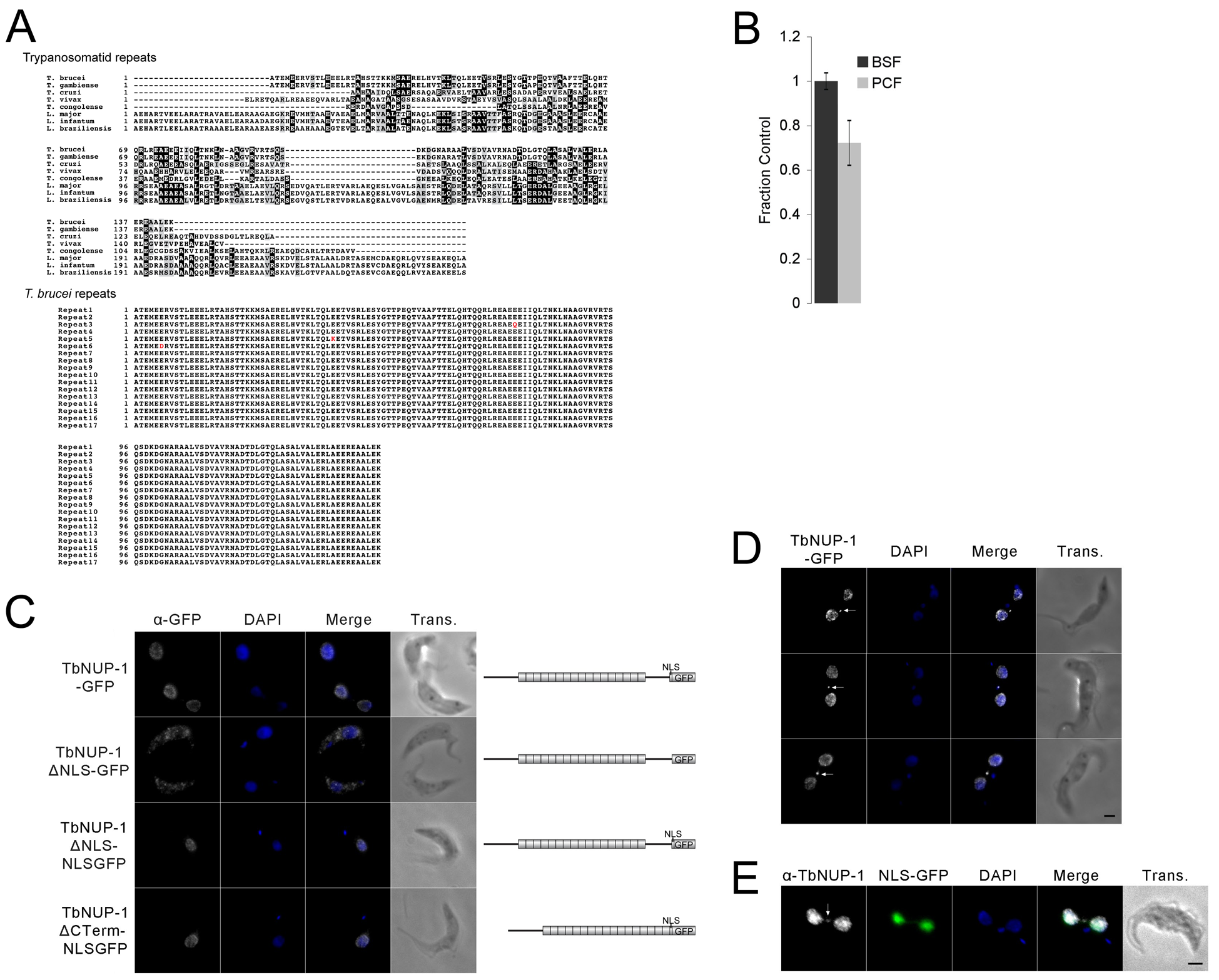

Supplement: Figure S1 — NUP-1 repeat structure, nuclear localization signal, and NUP-1 post-mitotic remnant. (A) Top: A single NUP-1 repeat from selected trypanosomatid species. Black boxes are identical residues and gray boxes similar in at least 50% of the included sequences. Lower: All of the repeats from T. brucei are identical with the exception of three conservative substitutions highlighted in red. (B) NUP-1 mRNA is present at similar levels in both BSF and PCF stages of T. brucei as measured by qRT-PCR and normalized to β-tubulin. (C) Wild type NUP-1-GFP (white) localizes to the nucleus (top panel). The nuclear localization is largely disrupted in cells expressing truncated NUP-1-GFP lacking the C-terminal 15 amino acids, which includes a predicted 11 amino acid nuclear localization signal (second panel). When the putative nuclear localization signal is added back onto the in situ tagging construct, nuclear localization is restored (third panel). When the C-terminus of NUP-1 is removed by a nuclear localization signal-containing tagging construct, NUP-1 still localizes to the nuclear periphery, suggesting that the C-terminal domain is not required for targeting (fourth panel). DAPI is used to visualize DNA (blue). Bar: 2 µm. (D) An additional localization was observed for NUP-1-GFP (white) between nuclei in late mitosis (anaphase) (white arrows). DAPI is used to visualize DNA (blue). Bar: 2 µm. (E) Cells expressing NLS-tagged GFP (green) and NUP-1-HA (white) were examined. It was found that NUP-1 localized to a slender connection between daughter nuclei in cells with incompletely segregated nuclei, possibly corresponding to a nuclear remnant remaining between daughter nuclei following mitosis derived from the midbody. DAPI is used to visualize DNA (blue). Bar: 2 µm. (TIF) [file pbio.1001287.s001.tif]

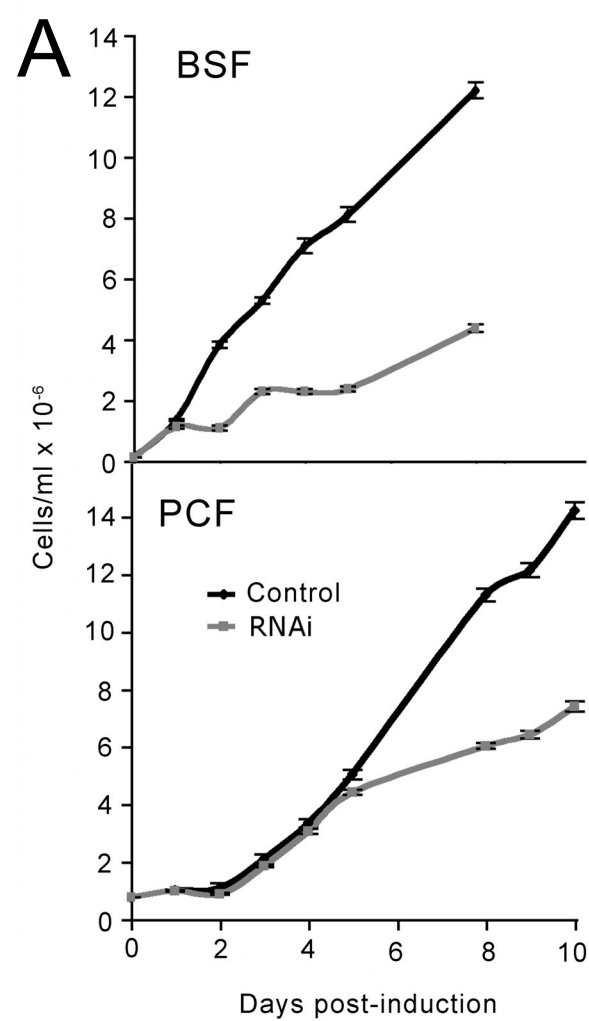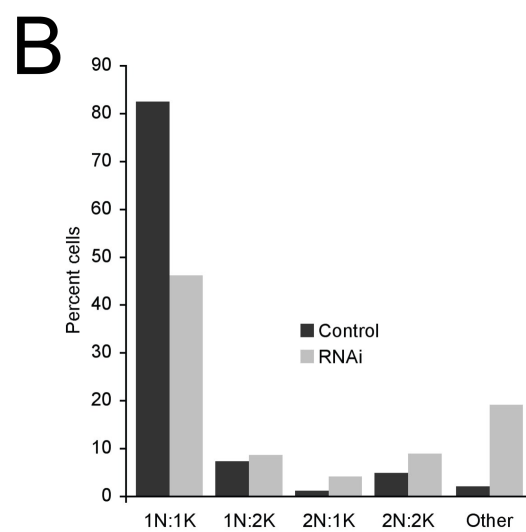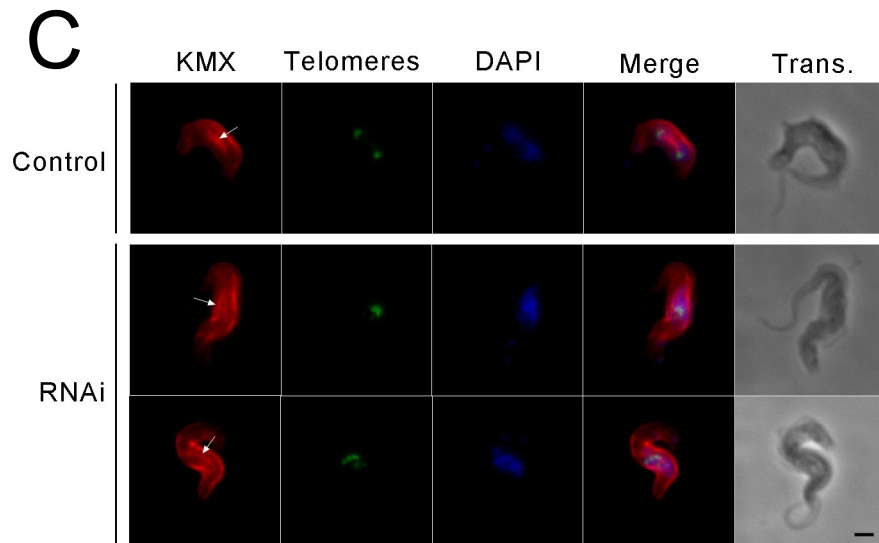

Supplement: Figure S2 — NUP-1 knockdown causes proliferative and NUP-1 lattice defects but does not affect spindle formation. (A) NUP-1 knockdown resulted in a significant growth defect compared to the control population in both BSF and PCF cells. (B) Cell cycle analysis of 200 BSF cells from control and RNAi populations 24 h after RNAi induction revealed a decrease in interphase cells (1N∶1K) and an increase in all other cell types observed. “Other” cells included those with nuclear and kinetoplast arrangements other than those scored in the remaining categories as well as nuclei that had abnormal protrusions and diffuse borders. (PDF) [file pbio.1001287.s002.pdf]

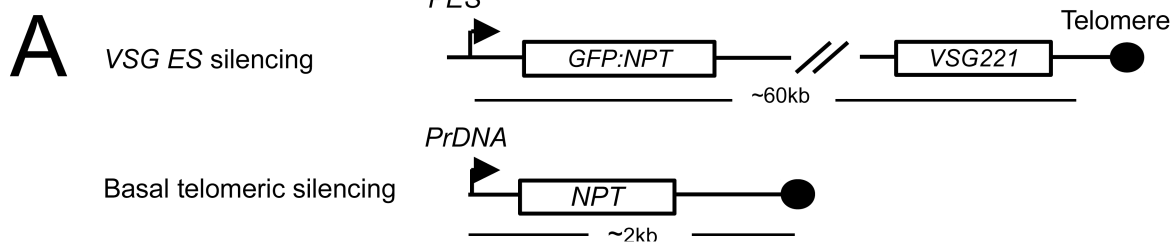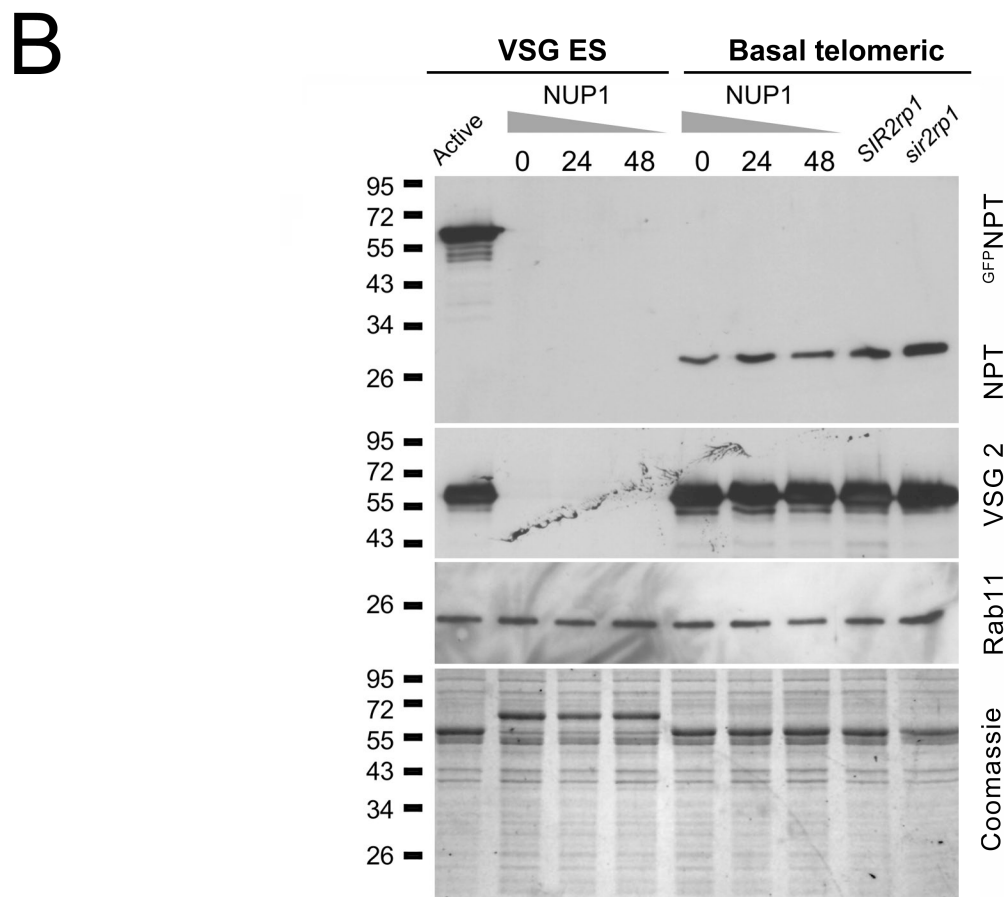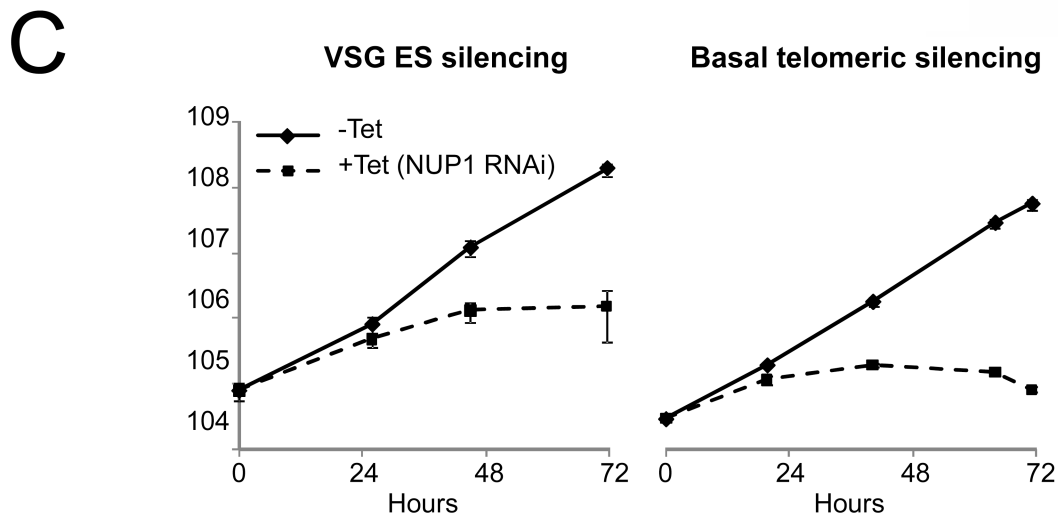

Supplement: Figure S4 — Protein levels from NUP-1 RNAi derepressed expression sites are not detectable by Western blot. NUP-1 depletion does not lead to protein levels from repressed sub-telomeric loci that are detectable by Western blot. The VSG 2 expression site and de novo telomere silencing cell lines (see Figure 9) were induced for 48 h. Expression of GFP:NPT or VSG 2 from the silenced BES1 is not detectable following RNAi against NUP-1; the parent cell line, where this expression site is active (“Active”), is shown for comparison. Similarly, no increase in NPT protein production was seen following NUP-1 RNAi when the reporter was located ∼2 kb from a de novo telomere; this reporter can be derepressed, as shown by the SIR2rp1 and sir2rp1 cell lines included. The Rab11 blot and the Coomassie stained gel serve as loading controls. NPT, neomycin phosphotransferase. (PDF) [file pbio.1001287.s004.pdf]

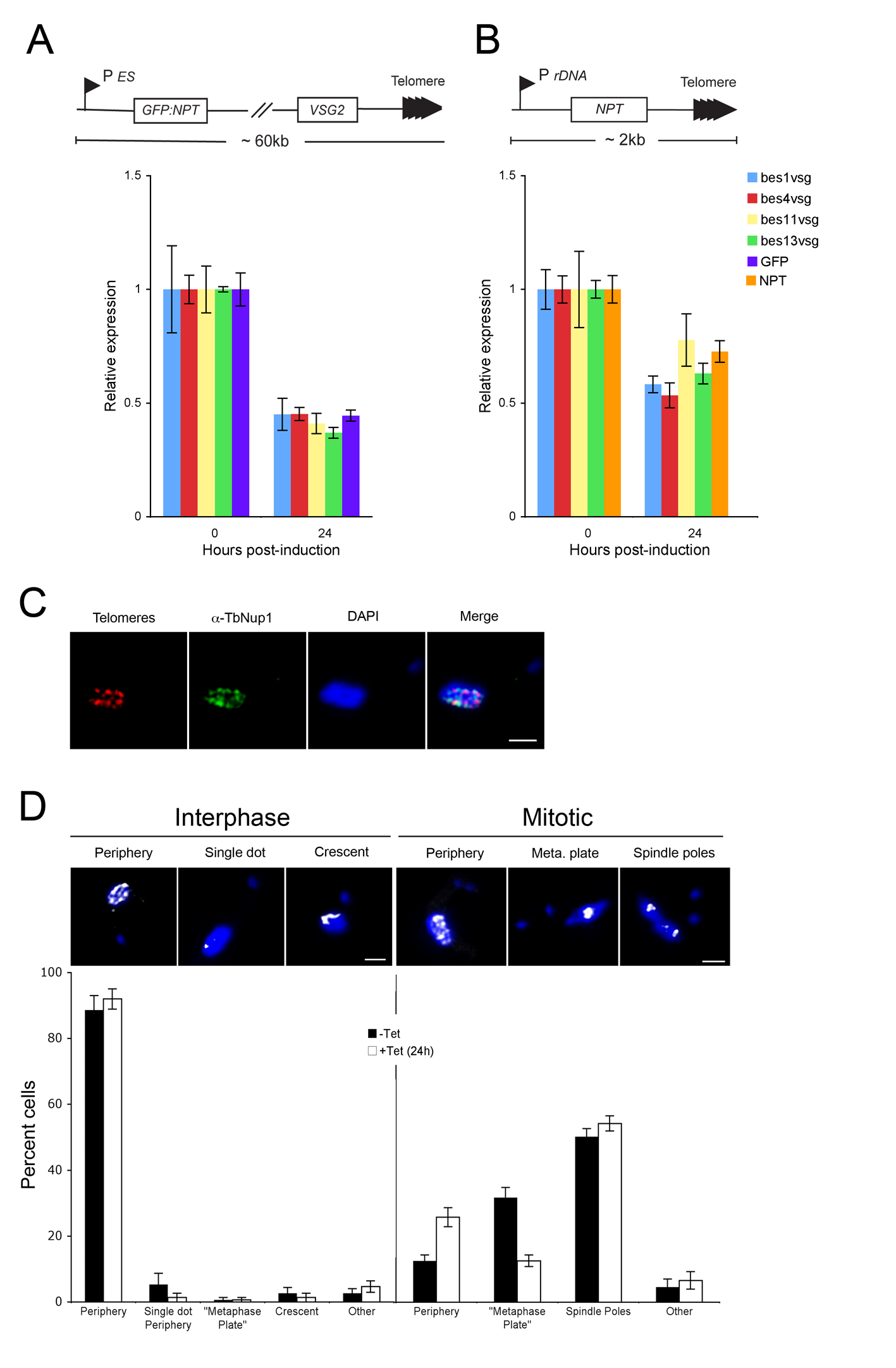

Supplement: Figure S5 — Depletion of Nup98 does not result in telomere mispositioning or misregulation of ES associated sub-telomeric genes. (A) Top panel: RNAi against TbNup98 was induced in cells containing a GFP::NPT reporter downstream of the repressed VSG2 ES promoter. Bottom panel: Expression of ES VSG genes and GFP::NPT reporter do not increase following depletion of TbNup98 as determined by qRT-PCR, normalised to Rab11. Note the difference between the behaviour here and in Figure 9 for NUP-1 knockdown. (B) Top panel: TbNup98 RNAi was induced in cells that contain an NPT reporter ∼2 kb upstream of a de novo telomere. Knockdown of TbNup98 resulted in a decrease in the expression of ES VSGs and the NPT reporter as determined by qRT-PCR, again distinct from the effect of NUP-1 RNAi. Knockdown of TbNup98 was validated by monitoring cell proliferation—doubling time was decreased by ∼50% at 24 h. (C) Cells were probed with anti-NUP-1 repeat region antibody (green) and FISH for telomeres (red). DAPI was used to visualise DNA (blue). The distribution of NUP-1 was unaffected by RNAi mediated knockdown of TbNup98. (D) Top panels: FISH images of telomeres in cells knocked down for TbNUP98. Following RNAi, the distribution of telomeres (white) was not substantially altered in interphase or mitotic cells. DAPI was used to visualise the DNA. Bars: 2 µm. Lower panels: Quantitative analysis of telomere positions comparing TbNup98 RNAi cells and uninduced control cells (+ and − Tet, respectively). Data showing the average and standard deviation derived from three independent TbNup98 RNAi cell lines are shown (n = 150 for uninduced and n = 150 for induced). There is no significant alteration to the telomere positioning or the migration of telomeres during cell division. (TIF) [file pbio.1001287.s005.tif]
