# Supplementary material for: NUP-1 Is a Large Coiled-Coil Nucleoskeletal Protein in Trypanosomes with Lamin-Like Functions
Source: PLoS Biol. 2012 Mar 27;10(3):e1001287. doi: 10.1371/journal.pbio.1001287 (PMC3313915; doi:10.1371/journal.pbio.1001287)
Supplement: Figure S3 — Control RNAi disrupts PCF proliferation and BSF NUP-1 RNAi increased expression of developmentally regulated genes. (A) RNAi induction of clathrin, polo-like kinase (PLK), and F0-F1 ATPase-associated factor (ATPaseAF) (right panel) all caused the expected growth defects in the reporter cell line. −, uninduced; +, induced. (B) Kinetics of procyclin and VSG gene expression changes in response to NUP-1 RNAi. Expression changes are on a logarithmic scale and relative to the expression level prior to RNAi induction and are derived from the array data shown in Figure 8. (C) Expression site VSG PCR products from qRT-PCR were sequenced (VSGXsequencing) and compared to the published bloodstream expression site VSG sequences (VSGX) and the array oligonucleotide from the 62 showing induction of expression most closely similar to the bloodstream expression site VSG (TbXoligo). Black boxes denote 100% sequence conservation. (PDF) [file pbio.1001287.s003.pdf]

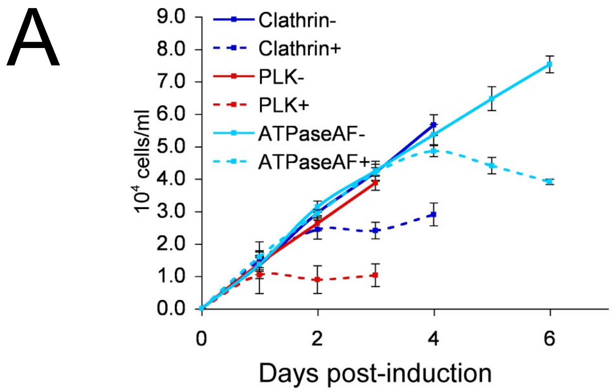

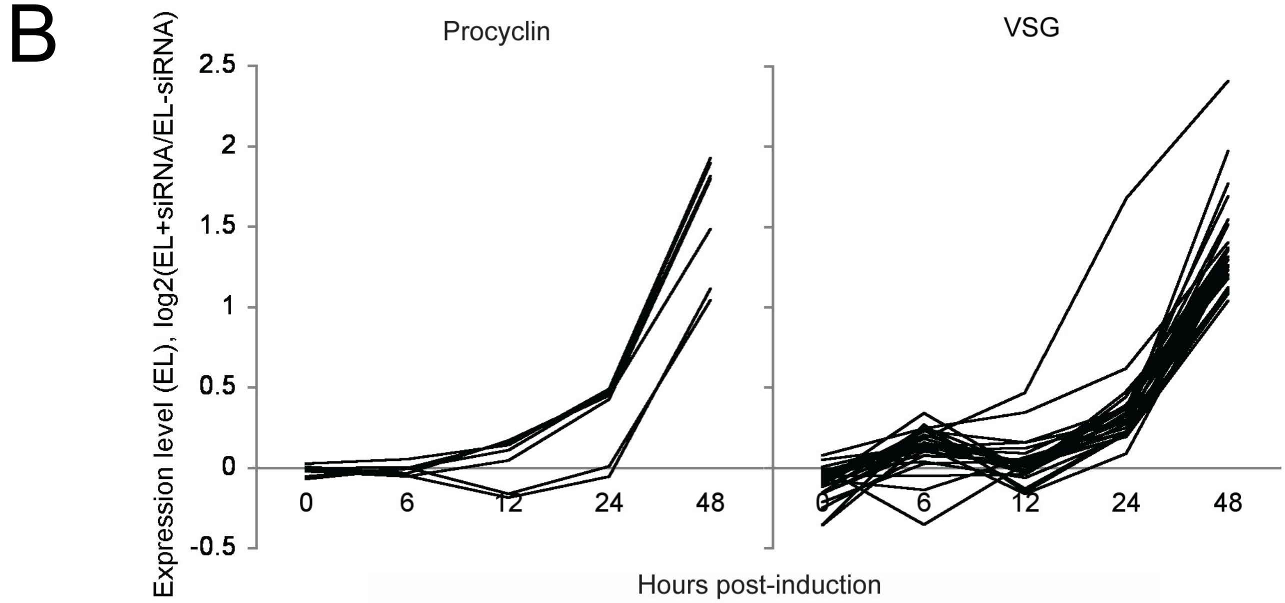

C

VSG 2

```
VSG 2 sequencing     45 TGCAAATCCCCATGCAAATGGCATAACGATGCGGAAAACAAAAAGTGCACATTGGATAAG
VSG 2               241 TGCAAATCCCCATGCAAATGGCATAACGATGCGGAAAACAAAAAGTGCACATTGGATAAG
Tb11.57.0084oligo     1 ---------GCAAATTGGTTGAAGAAGAAGGTGGCAAA-AAGAAGTGCACATTGGATGCA

VSG 2 sequencing    105 GAGGAGGCAAAAAAGGTAGCAGATGAGACTGCAAAAGATGGGAAAACTGGAAACACAAAC
VSG 2               301 GAGGAGGCAAAAAAGGTAGCAGATGAGACTGCAAAAGATGGGAAAACTGGAAACACAAAC
Tb11.57.0084oligo    51 AAAGCAGCTA--------------------------------------------------
```

VSG 21

```
VSG 21              241 CAAAGTTGATACTGAGAAAAAAGATGACAAATCGTTTTCTAGCAATGTGAGAGTGTCTGT
VSG 21 sequencing   108 CAAAGTTGATACTGAGAAAAAAGATGACAAATCGTTTTCTAGCAATGTGAGAGTGTCTGT
Tb09.244.0640oligo    1 ----------------------------------------AGCAATGTGAGAGTGTCTGT

VSG 21              301 TCCTCAGGTATTTGCTGCGTTAGTTTTGGCTGCATTCTAAGAATTCTGAC
VSG 21 sequencing   168 TCCTCAGGTATTTGCTGCGTTAGTTTTGGCTGCATTCTAAGAATTCT---
Tb09.244.0640oligo   21 TCCTCACGTGTTTGCTGCATTTGCTGCC-TTGCTTTTTAA----------
```

VSG 16

```
VSG 16               61 ATCAGAGAAGTCAACTTCCGAAGCAGACGTAACGTGCGAAAAGAAAGGAACAGGAGATAA
VSG 16 sequencing     1 --------------CTTCCGAN-CANACGTAACGTGCGAAA-GAAAGGAACAGGAGATAA
Tb927.4.5530oligo     1 ----------------------------------------------------------GC

VSG 16              121 CTGCAAAGATGGATGTAAAGAAGTCGAAGAAGGTGGAAAAAAGAAGTGCGTAAAGGATCC
VSG 16 sequencing    45 CTGCAAAGATGGATGTAAAGAAGTCGAAGAAGGTGGAAAAAAGAAGTGCGTAAAGGATCC
Tb927.4.5530oligo     3 TTGCAAAGATGGCTGCAAAGAAATCAGTGAAAACGGCCAAAAGAAGTGCGTAGTGGAT--
```

VSG 17

```
VSG 17              241 TAAAACAAACACCACAGGAAGTAATTCTCTTGTGATTAAAAAGGCGCCTCTTTTGCTTGC
VSG 17 sequencing   142 TAAAACAAACACCACAGGAAGTAATTCTCTTGTGATTAAAAAGGCGCCTCTTTTGCTTGC
Tb09.244.1790oligo    1 ----------GGAGCAGTTGTTATTTCTGCTGTGATTAA---TGCCCCTCTTTTGCTTGC

VSG 17              301 ATTTTTGATTCTAGCATAACTTTCCCCCTC------------------------------
VSG 17 sequencing   202 ATTTTTGATTCTAGCATAACTTTCCCCCTCATATTTCCCCTCTTTTTAAAATTTTCCTTG
Tb09.244.1790oligo   48 ATTTTTGATTCTA-----------------------------------------------
```
