# Supplementary material for: NUP-1 Is a Large Coiled-Coil Nucleoskeletal Protein in Trypanosomes with Lamin-Like Functions
Source: PLoS Biol. 2012 Mar 27;10(3):e1001287. doi: 10.1371/journal.pbio.1001287 (PMC3313915; doi:10.1371/journal.pbio.1001287)
Supplement: Table S1 — NUP-1 orthologues in trypanosomatids. Accession numbers of sequences with homology to NUP-1. In species indicated by multiple accession numbers, the NUP-1ORF appears to have been split into multiple parts during genome annotation and assembly. (PDF) [file pbio.1001287.s012.pdf]

Species	Accession number
Trypanosoma brucei	Tb927.2.4230
Trypanosoma gambiense	Tbg.972.2.2350, Tbg.972.2.2360
Trypanosoma cruzi	AAS44545.2, Tc00.1047053511617.9, Tc00.1047053509099.5
Trypanosoma vivax	TvY486_0201120, TvY486_0039210
Trypanosoma congolense	TcIL3000.2.710
Leishmania major	LmjF27.1820
Leishmania infantum	LinJ27_V3.1720
Leishmania braziliensis	LbrM27_V2.1960
