# Supplementary material for: NUP-1 Is a Large Coiled-Coil Nucleoskeletal Protein in Trypanosomes with Lamin-Like Functions
Source: PLoS Biol. 2012 Mar 27;10(3):e1001287. doi: 10.1371/journal.pbio.1001287 (PMC3313915; doi:10.1371/journal.pbio.1001287)
Supplement: Table S2 — Percent identity/similarity for the alignment of full-length NUP-1 with other trypanosomatid NUP-1 sequences. Percent identity is shown in the lower portion of the table and percent similarity in the upper portion. NUP-1 is most similar to the sequence from T. b. gambiense, which is essentially a subspecies of T. brucei. Homology is decreased amongst other African trypanosomes and lower still for the South American trypanosome T. cruzi. There is very low identity/similarity between NUP-1 and putative orthologues in Leishmania. However, the NUP-1 gene remains syntenic in these species. (PDF) [file pbio.1001287.s013.pdf]

	T. brucei	*T. gambiense*	*T. cruzi*	*T. vivax*	*T. congolense*	*L. major*	*L. infantum*	*L. braziliensis*
T. brucei		100	59.76	50	43.35	13.67	14.06	14.45
T. gambiense	98.82		59.76	50	43.35	13.67	14.06	14.45
T. cruzi	51.17	51.56		50.39	54.29	21.48	23.04	21.09
T. vivax	43.75	44.14	45.7		54.29	17.57	18.75	18.75
T. congolense	35.54	35.54	46.87	48.04		18.75	20.7	19.92
L. major	8.98	8.98	11.32	10.93	10.93		94.92	83.2
L. infantum	9.76	9.76	12.5	10.93	11.32	92.96		85.15
L. braziliensis	8.98	8.98	11.32	11.32	10.93	75.39	75.39	
